# Errors in visual search: Are they stochastic or deterministic?

**DOI:** 10.1186/s41235-024-00543-z

**Published:** 2024-03-19

**Authors:** Aoqi Li, Johan Hulleman, Jeremy M. Wolfe

**Affiliations:** 1 The University of Manchester, Manchester, UK; 2 Brigham and Women’s Hospital, Boston, MA USA; 3 Harvard Medical School, Boston, MA USA

**Keywords:** Visual search, Deterministic error, Stochastic error

## Abstract

In any visual search task in the lab or in the world, observers will make errors. Those errors can be categorized as “deterministic”: If you miss this target in this display once, you will definitely miss it again. Alternatively, errors can be “stochastic”, occurring randomly with some probability from trial to trial. Researchers and practitioners have sought to reduce errors in visual search, but different types of errors might require different techniques for mitigation. To empirically categorize errors in a simple search task, our observers searched for the letter “T” among “L” distractors, with each display presented twice. When the letters were clearly visible (white letters on a gray background), the errors were almost completely stochastic (Exp 1). An error made on the first appearance of a display did not predict that an error would be made on the second appearance. When the visibility of the letters was manipulated (letters of different gray levels on a noisy background), the errors became a mix of stochastic and deterministic. Unsurprisingly, lower contrast targets produced more deterministic errors. (Exp 2). Using the stimuli of Exp 2, we tested whether errors could be reduced using cues that guided attention around the display but knew nothing about the content of that display (Exp3a, b). This had no effect, but cueing all item locations did succeed in reducing deterministic errors (Exp3c).

**Supplementary Information:**

The online version contains supplementary material available at 10.1186/s41235-024-00543-z.

## Introduction

Individuals routinely fail to report or respond to visual stimuli that are clearly visible, “right in front of their eyes”. This is unfortunate if the stimulus is a typo in your CV. It is markedly more serious if it is a tumor in a chest x-ray. The nature of these errors is important, not the least because it can have legal consequences, in the case of the tumor, if not the typo (Berlin & Hendrix, 1998; Duszak & Robinson, 2022). How we or a court may think about an error may depend on whether it is stochastic, occurring randomly, or deterministic, occurring any time a target appears in a specific location in a particular scene. In this paper, we describe a method for categorizing the type of error as stochastic or deterministic and we consider possibilities for mitigation.

In some cases, a clearly visible, missed item is an unexpected item. The Simons and Chabris (1999) gorilla is the most famous example of such “inattentional blindness” (Koivisto et al., 2004; Kuhn & Tatler, 2011; Mack & Rock, 1998; Macknik et al., 2008; Simons, 2000; Simons & Chabris, 1999). Inattentional blindness has been invoked as an explanation for some real-world errors; for example, how a driver may fail to notice an unexpected road user before a road accident or, more benignly, how an audience member may be induced to believe that something has materialized from nothing in a magic show. Some researchers have claimed that such “inattentional blindness” involves attentional misdirection in magic or elsewhere. The observer is blind because the magician or the situation has moved the observer’s attention away from the critical event (Barnhart & Goldinger, 2014; Kuhn & Tatler, 2005; Kuhn et al., 2008). Other researchers have proposed that a failure to see some highly noticeable objects is due to an illusion that the space behind an occluding foreground object is experienced as empty (“the illusion of absence”, Ekroll et al., 2021). Based on the assumption that the region is empty, the observer may fail to note an item even when movement of the occluder or the observer makes the target visible. Ekroll et al (2021) proposed this illusion as an important contributor to 'looked-but-failed-to-see' (LBFTS) errors in driving situations where an item, hidden by a car’s ‘blindspot’ is not seen even when movement of the car makes the item visible.

Missed gorillas and other examples of inattentional blindness are dramatic but they are far from the only type of LBFTS error (Wolfe et al., 2022). Clearly visible targets are routinely missed even when the searcher knows that these targets, be they typos or tumors, are relevant to their ongoing task. In typical LBFTS driving accidents, the driver will generally know that they should be watching for pedestrians, turning vehicles, etc. (Pamme et al., 2018). In medical settings, when a clinician fails to report an “incidental finding”, it will not be a missed gorilla (Drew et al., 2013). It is more likely to be a secondary, but clinically significant finding that the clinician knows might occur in this setting (Lumbreras et al., 2010). Indeed, a missed item can be the actual target of a search (Hovda et al., 2022, 2023). Medical errors by radiologists are an example. Clinicians will sometimes miss targets like pulmonary nodules even if they are clearly visible when pointed out. Kundel et al (1978) classified such errors into three groups based on eye tracking data. The three groups are search, recognition and decision errors. These remain widely used in the analysis of errors. An error is deemed to be a search error when the target (e.g. a lung nodule) never falls within a "functional visual field” surrounding that target (Sanders, 1970; Wolfe et al., 2021). Recognition errors occur when the eyes fixate on or near the target but the eyes move on without the observer having apparently noted the targets presence. These can be classified as a type of LBFTS errors. Decision errors occur when the observer spends significant time looking at or near the target but still does not label it correctly. In this case, the observer did not fail to see but misclassified the item. In breast radiology, perhaps 70% of missed lesions on mammograms are retrospectively visible when pointed to, after the fact. The search-recognition-decision taxonomy can classify those errors, given eye tracking data but classification is not explanation. Many different factors could underly the errors, including satisfaction of search, incorrect background sampling, and incorrect first impressions (Gandomkar & Mello-Thoms, 2019). We are seeking to understand if these and other factors operate at chance or whether some configurations of stimuli are more error prone. Moreover, we also aim to investigate ways of reducing those errors, even if it is unlikely that there is a general method to reduce all kinds of errors.

In the present work, we are using a simple letter search task. However, even in a very basic laboratory visual search task like a search for a perfectly visible “T” among other distractor letter, "L"s, observers will routinely miss 5–10% of targets. When targets are missed, are those errors random (henceforth “stochastic”)? That is, if participants miss, let us say, 10% of targets, is that a random set of 10% of all target trials, or are observers more likely to miss some specific targets in some specific displays? In the limit, would participants miss the same targets again, if asked to search the same displays? We will call such errors “deterministic”. In addition to examining the nature of the errors, this paper also tests several cueing interventions to see if they can reduce errors and which errors can be reduced. To categorize the errors, a set of T among L search displays was presented twice to each participant. We calculated the miss rate, $P1$, for the first time that the set of displays was shown and, $P2$, for the second time. We also calculated the proportion cases where both copies were missed: $P12$. If the errors are stochastic, then $P12=P1*P2$. If the errors (on the first or the second copy) are deterministic, $P12={\text{min}}(P1, P2)$. If errors are a mix of stochastic and deterministic, $P12$ will fall between these two predictions. In addition to the analysis on the qualitative nature of these errors, it is possible to calculate the relative proportions of stochastic and deterministic errors, based on the three observable quantities: $P1$, $P2$ and $P12$. This calculation allowed us to evaluate the effect of the cueing interventions. If an intervention was useful, did it reduce stochastic or deterministic errors? If these interventions reduce errors on a simple T-vs-L search task, it might be worth trying a similar strategy in socially important, real-life tasks.

## Experiment 1: basic search for a T among Ls

Experiment 1 consisted of a simple visual search task where white letters were presented against a gray background.

### Participants

The experiment was run online on the Pavlovia platform (https://pavlovia.org). When recruiting participants for Experiment 1 and all the subsequent experiments, we didn’t set the language filter to ensure participants understand the instructions, but we set practice trials and excluded participants based on d’ after we got the data. Therefore, participants who don’t understand the instructions and are guessing will not be included. The only additional filters we set are age (18–100), vision (yes) and exclude participants from previous studies (this criterion applies to experiments after Exp 1). For Experiment 1, we tested 20 participants (6 males, 14 females, mean = 19.5, SD = 0.9, min = 18, max = 21) from the BSc Psychology programme at the University of Manchester. All participants reported normal or corrected-to-normal vision and gave their informed consent before they began the experiment. Participants received course credit for their participation. Ethics approval came from The University of Manchester (2023–16117-27,175).

### Stimuli & apparatus

**Fig. 1:**
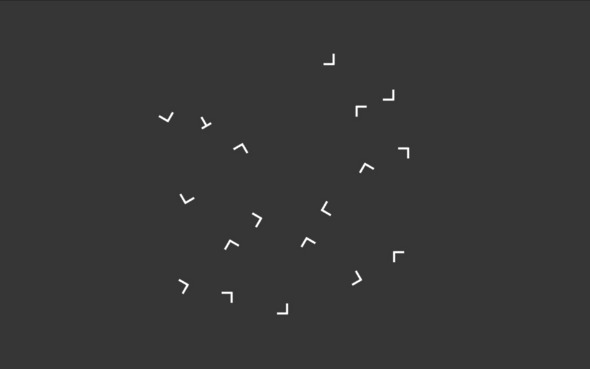
Sample stimulus for Experiment 1

The experiment was programmed in Python and translated into javascript by PsychoPy (Peirce et al., 2019). The online version was hosted on Pavlovia. Figure 1 shows the stimuli for Experiment 1. They consisted of an array of white letters (T and Ls) against a gray background. The length of vertical and horizontal line segments of the Ts and Ls was 0.03 screen height (note that because we were testing on-line, we had relative, not absolute control of the sizes of stimuli). The orientations of the letters were randomly and uniformly selected from rotations of 30, 60, 90, 120, 150, 180, 210, 240, 270, 300, 330, & 360 deg. The positions of the letters were randomly generated for each trial such that all items fit in a square region that had a side length of 0.7 screen height, centered on the middle of the screen. In addition, the minimum distance between any two letters was always larger than 0.1 screen height.

### Design & procedure

Participants searched for the letter T among Ls. Participants were instructed to press ‘j’ if they found the target and ‘f’ if they did not. The stimulus was present until response. After the initial response, participants could press the space bar within one second to reverse the response if they thought they made a motor error. Targets were present on 50% of trials. Trial by trial feedback was not given, but after every block of 100 trials the proportion correct for that block was displayed. There were two set sizes; 18 and 36, fully crossed with target presence and target absence. For each participant, we generated 75 versions of each of the four resulting combinations for a total of 300 unique stimulus displays. Each of these was presented twice. The two copies of the 300 stimuli were randomly intermixed across six blocks of 100 trials for a total of 600 trials for the experiment (Please note that this means that the minimum distance between two copies could be 1, i.e., when they were presented in consecutive trials, whereas the maximum distance could be 599, i.e., first copy presented in trial 1, second copy presented in trial 600). Thus, there were three factors in this design, each with two levels: repetition (first, second), set size (18, 36), and target (present, absent). Participants completed four practice trials before they started the experiment.

### Analysis method

We focused on the RT data and miss rate data, with our primary interest being in the miss rate data. The RT data was subjected to a three-way repeated measure ANOVA with target presence, set size and repetition as within-subject factors. Since the experiment involved a typical visual search task, we found the typical main effects of target presence and set size. Specifically, there were longer reaction times for absent trials and longer reaction times for larger set sizes. A two-way interaction between target and set size, showing steeper reaction time slopes for absent trials, also occurred. All of these effects are highly statistically reliable and will not be reported in detail in the Results section. The results of the full ANOVAs are shown in supplementary tables.

**Fig. 2:**
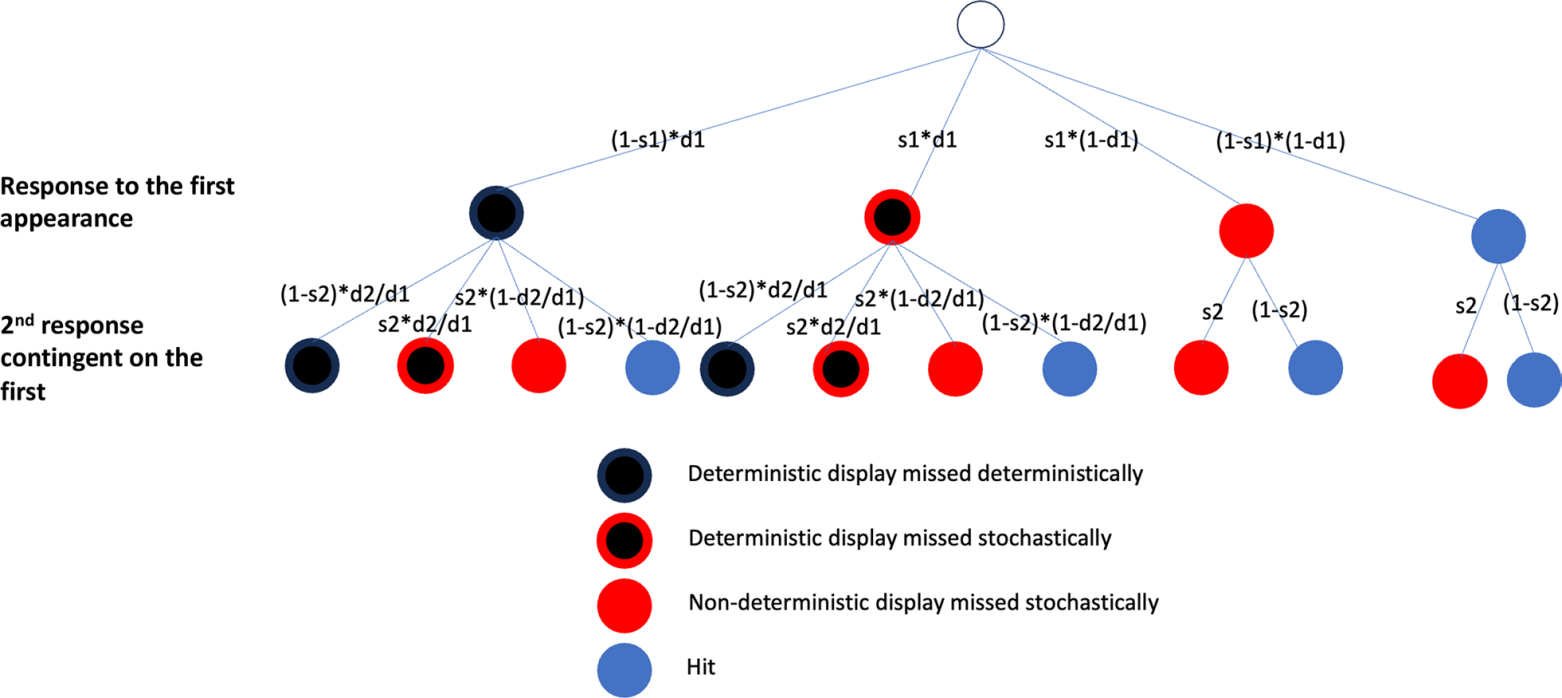
Observed errors decomposed into deterministic errors and stochastic errors

For miss rate data, we calculated the miss rate, $P1,$ for the first time the set of displays was shown and, $P2,$ for the second time. We also calculated the proportion of cases where both copies were missed, $P12$. If the errors are stochastic, then $P12=P1*P2$. If the errors (on the first or the second repetition) are deterministic, $P12={\text{min}}(P1,P2)$. If the errors are a mix of stochastic and deterministic errors, $P1*P2<P12<{\text{min}}(P1, P2)$. To get a quantitative estimate of the relative proportion of stochastic and deterministic errors, we modelled how the errors observed in round 1 and round 2 could be decomposed into different types of error, as shown in Fig. 2. One complication worth noting is that a deterministic display may produce a stochastic error. A deterministic display is one that would produce a deterministic error. However, it is possible for an error to be produced on that trial for stochastic reasons. Imagine, for instance, that the observer is simply not paying attention on that would-be deterministic trial and pushes a response button at random.

In Fig. 2, there are four possible states for a trial.^1^ The target in a trial is either fundamentally unfindable (black) or fundamentally findable (non-black). A completely black circle represents the case where a deterministic error is made on a trial with an unfindable target. A black circle with a red border means that the unfindable target was missed stochastically. A solid red circle represents the situation where a stochastic error is made on a trial with a findable target. A blue circle represents a trial where the target is successfully found. If the target in a trial is fundamentally findable, this target cannot become fundamentally unfindable. This means that it is not possible to transition from a non-black circle to a black circle or a black circle with a red border. A transition in the opposite direction is possible though. For instance, if a cueing intervention works and reduces the number of deterministic errors, it is possible for a deterministic miss (black) on one trial to become a hit (blue) on its next appearance.

To describe the proportions of deterministic and stochastic errors, four parameters are introduced: $d1$ and *d*2 represent the proportion of deterministic errors relative to the total number of stimuli in round 1 and round 2. $s1$ and $s2$ represent the stochastic error rates for a stimulus in round 1 and round 2 respectively. In Fig. 2, *Row 0* with an empty circle represents the to-be-determined status of one trial. *Row 1* with four different types of circles represents the four possible outcomes of the first appearance of a trial with the notation for the corresponding probabilities in round 1. *Row 2* represents the possible outcomes with the notation for the corresponding probabilities in round 2. Therefore, the observed $P1, P2$ and $P12$ can be theoretically decomposed into the summed error probabilities in round 1 and round 2. The following three equations can be derived (The original versions and the simplifying process can be found in the appendix):

$$P1=d1*\left(1-s1\right)+s1$$

$$P2=d2+s2*\left(d1-d2\right)+\left(1-d1\right)*s2$$

$$P12=d2+s2*\left(d1-d2\right)+s1*\left(1-d1\right)*s2$$

For Experiment 1, there was no cueing intervention. A fixed deterministic rate was therefore assumed for round 1 and round 2, i.e., $d=d1=d2$. With this additional assumption, there is a unique solution for the above equations.

$$d=\frac{P12-P1*P2}{1-P1-P2+P12}$$

$$s1=\frac{P1-P12}{1-P2}$$

$$s2=\frac{P2-P12}{1-P1}$$

### Data exclusion

Since the data were collected online, we first checked if there were extreme long RTs (larger than 100 s) that might cause large shifts in means and standard deviations and excluded those extreme long RTs to calculate the limit of mean $\pm$ 2.5 SD. Then trials with RTs smaller or greater than 2.5 SD from the mean RT in each cell of the combination target x set size (3.47%) and trials where participants corrected their motor responses (1.07%) were removed for each observer. When one trial was removed, the other copy of the trial was also be removed (93.3% remained). After the removal of the above trials, we further checked the d’ of all the participants. Participants with d’ beyond 2.5 SD from the group mean for each individual experiment were excluded. One participant with a low d’ = 1.02 was removed from Exp 1. For the remaining participants, min d’ = 2.95, max d’ = 5.84.

## Results

### RTs

**Fig. 3:**
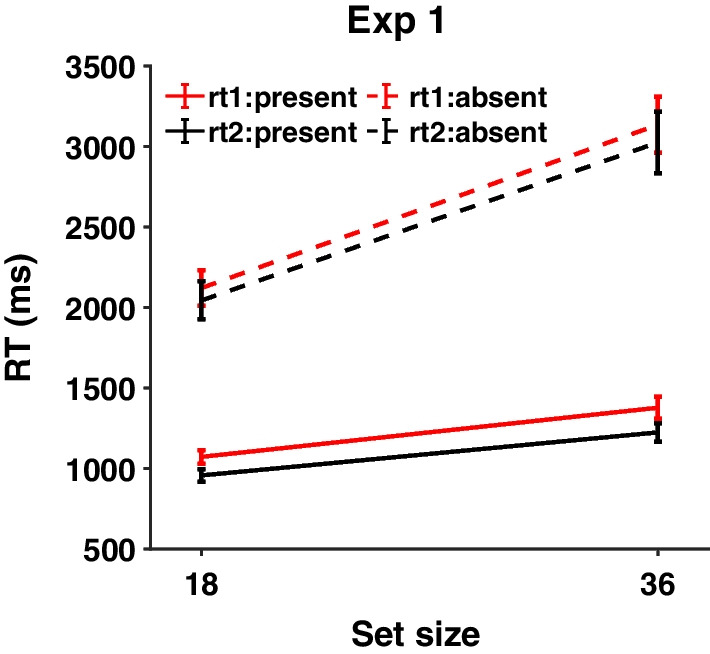
RTs from Experiment 1 as a function of set size, target presence, and repetition. Red lines: first presentation, Black lines: second presentation. Full lines: present trials, Dotted lines: absent trials. Error bars represent $\pm 1$ SEM

Figure 3 shows RTs on correct response trials for Experiment 1. It is clear that the first and second repetitions of the stimuli produce very similar RTs with a slight speed-up on the second appearance. The three-way repeated measure ANOVA with target presence, set size and repetition as within-subject factors shows a main effect of repetition [F(1, 18) = 7.00, *p* = 0.016, ${\eta }_{p}^{2}$ = 0.28], suggesting that participants responded faster in round 2 than in round 1. The interaction between target presence and repetition [F(1, 18) = 0.37, *p* = 0.55, ${\eta }_{p}^{2}$ = 0.02] as well as the interaction between set size and repetition [F(1, 18) = 0.50, *p* = 0.49, ${\eta }_{p}^{2}$ = 0.03] was not significant. The three-way interaction among all the factors was not significant either [F(1, 18) = 0.004, *p* = 0.95, ${\eta }_{p}^{2}$ = 0.00]. The full results of the three-way ANOVA are presented in Additional file 1: Figure S1. Individual RTs are given in Additional file 1: Table S1.

### Miss rates

**Fig. 4:**
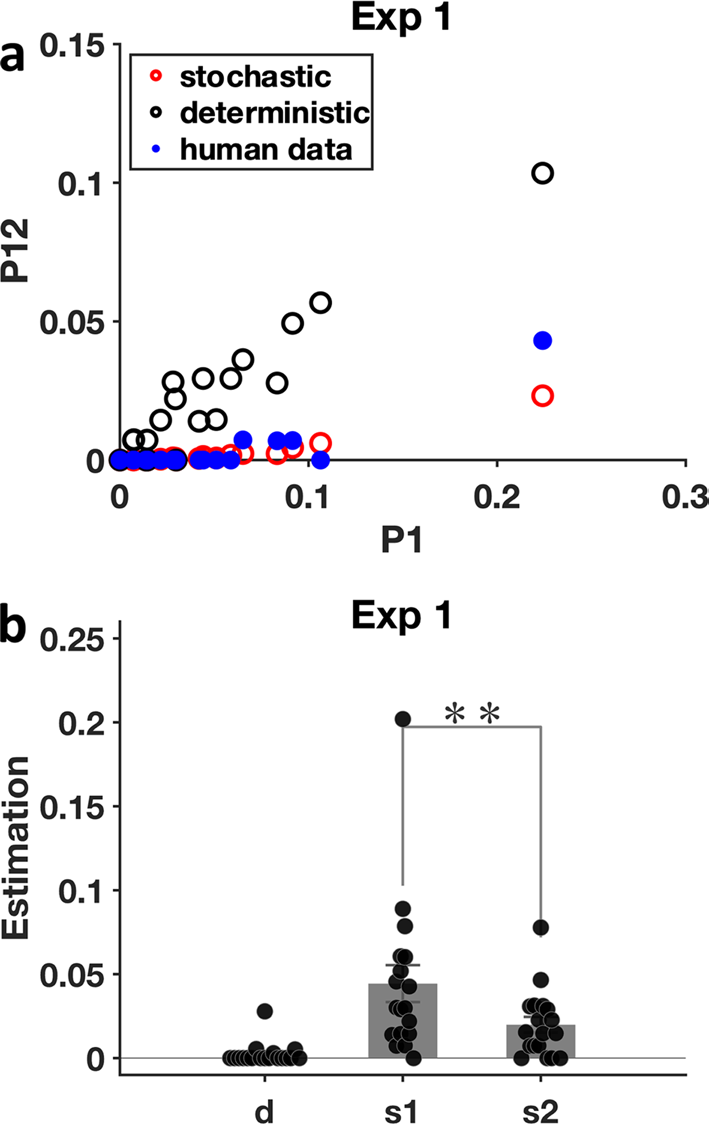
Miss rate analyses for Experiment 1. **a** Comparison between observed human data, stochastic predictions and deterministic predictions for each observer. **b** Deterministic error proportion $d$ and stochastic error rates $s1$ and $s2$ calculated from human data. Error bars represent $\pm 1$ SEM

Figure 4 shows the results of the miss rate analyses for Experiment 1. In the scatter plot in Fig. 1, the proportion of targets missed twice (P12) is plotted as a function of the proportion of targets missed on the first appearance. The blue dots represent the observed data for each participant with $x=P1$ and $y=P12$ calculated from human data. Each observed data point (blue dot) is paired with the stochastic prediction (red circle) and the deterministic prediction (black circle) of $P12$ given the observed $P1$ and $P2$. Therefore, for one participant with observed $P1$, $P2$ and $P12$, each observed data point (blue dot) is $(P1, P12)$, the stochastic prediction (red circle) is $(P1, P1*P2)$, and the deterministic prediction (black circle) is $(P1,\mathrm{ min}(P1,P2))$. As can be seen, the observed data (blue dots) are almost overlapping with the stochastic predictions (red circles). The bar plot in Fig. 1 shows the results of the parameters solved using the equations from the method section, above. The figure is based on the assumption that $d=d1=d2$, resulting in three parameters to be computed i.e., the deterministic error proportion, $d$, the stochastic rate in round 1, $s1,$ and the stochastic rate in round 2, $s2$. $d$, $s1$, $s2$ were all supposed to be positive rates (including 0), so one participant with a calculated $d$ smaller than $-0.002$ was excluded. For other participants, when -$0.002 \le d < 0$, *d* was rounded to 0. A one-sample t-test showed that the deterministic error proportion $d$ was not significantly different from 0 [t(17) = 1.47, *p* = 0.159, Cohen’s d = 0.35], demonstrating that errors in Experiment 1 were almost exclusively stochastic. A paired t-test comparing the stochastic rates, $s1$ and $s2$, shows that observers made fewer stochastic errors in round 2 than in round 1 [t(17) = 3.48, *p* = 0.003, Cohen’s d = 0.82], indicative of some learning effect over the course of Experiment 1.

### Experiment 1 discussion

Experiment 1 consisted of a simple T-vs-L search task where all the white letters were presented on a gray background. Analyses of the RTs and miss rates showed that observers responded faster and made fewer errors in round 2 than in round 1, indicative of some learning effect. Learning effects could include both an effect of time on task (participants perform better or worse over time due to practice or fatigue) and a repetition effect (participants may know something about the stimulus when they encounter it for the second time), but this is not relevant to our aim of categorizing errors as deterministic or stochastic. More importantly for present purposes, the proportion of deterministic errors, $d,$ calculated from miss rates was not significantly different from 0, indicating that errors were almost purely stochastic in this experiment. The result would be different if there was a systematic bias in search. For example, if observers tended to ignore the lower left corner of the display, then targets in the lower left would be more likely to be missed on both their first and second appearances. This is not what is found with these simple and clear stimuli. However, in many real-world search tasks (mammography, airport security), search items are not so clearly visible. In the next two experiments we therefore tested whether stochastic errors still dominate when items become harder to distinguish from the background.

## Experiments 2a and 2b: letters on a noisy background

In Experiments 2a and 2b, the uniform gray background was replaced by a noisy background. The letters were also of various grayscales. The only difference between the two experiments was that Experiment 2b used a more restricted set of target contrasts and target locations compared to Experiment 2a.

### Participants

Both Experiments 2a and 2b were run online on the Pavlovia platform (https://pavlovia.org). For Experiment 2a, we tested 21 participants (6 males, 13 females, mean = 23.1, SD = 7.1, min = 18, max = 45, two participants did not provide the gender and age information). Thirteen of them were from the BSc Psychology programme at the University of Manchester and eight of them were recruited via Prolific. For Experiment 2b, we tested 21 participants (8 males, 8 females, 1 non-binary, mean = 28.8, SD = 10.9, min = 20, max = 57, four participants did not provide the gender and age information) recruited via Prolific. All participants reported normal or corrected-to-normal vision and gave their informed consent before they began the experiment. Participants received course credit (when recruited from the BSc Psychology) or 8 GBP (when recruited via Prolific) for their participation. Ethics approval came from The University of Manchester (2023–16117-27,568 [credit version] and 2023–16117-28,440 [payment version] for Exp 2a, 2023–16117-28,499 for Exp 2b).

### Stimuli & apparatus

In Experiments 2a and 2b, the stimuli consisted of an array of T and Ls against a background composed of $1/{f}^{1.3}$ noise. The noise was intended to roughly simulate the texture of a mammogram. Ts and Ls were of various grayscales. The length of vertical and horizontal lines of Ts and Ls was 0.03 screen height. The orientations of the letters were randomly selected from [30, 60, 90, 120, 150, 180, 210, 240, 270, 300, 330, 360]. The minimum distance between any two letters was always larger than 0.1 screen height to avoid overlapping.

In Experiment 2a, The grayscales for items were randomly generated by the formula (rand()-0.5)*2 for each trial. Therefore, in Experiment 2a, the distribution of item grayscale values was uniform. The default colour space in PsychoPy ranges from -1 to 1, so the recorded values were converted to 0–255 for subsequent analyses. The positions of the letters were randomly generated for each trial with the limitation that both x and y ranged from [0.15, 0.85] screen height. The noisy background was randomly selected from 10 noise images of 2000*2000 pixels for each trial and was cropped from the centre to fit the screen size during the online testing. Therefore, Experiment 2a required participants to use a screen smaller than 2000*2000.

**Fig. 5:**
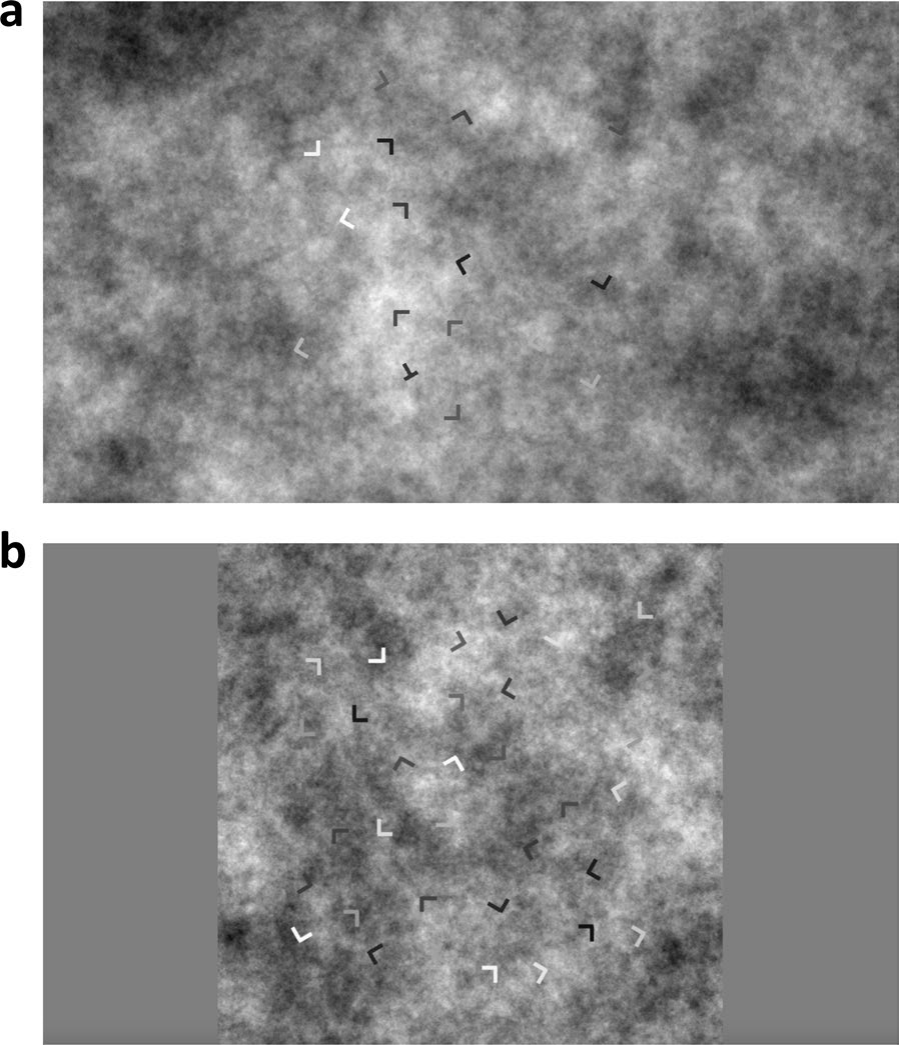
Sample stimuli from Experiments 2a and 2b. **a** In 2a, the background filled the entire screen. **b** In 2b, the background was a square with size determined by the vertical extent of the screen

In Experiment 2b, the target contrast (defined by the difference between target grayscale and background grayscale [T-B]) was controlled to be [− 105, − 75, − 45, − 15, 15, 45, 75, 105] and the locations of the target were evenly distributed across four spatial quadrants (upper left: x, y from [0.15, 0.45]; upper right: x from [0.55, 0.85], y from [0.15, 0.45]; bottom left: x from [0. 15, 0.45], y from [0.55, 0.85]; bottom right: x, y from [0.55, 0.85]). To achieve this manipulation, we generated the stimuli before the experiment. Crossing target presence (2), set size (2), T-B (8) and target location (4) yielded 128 combinations (2*2*8*4). Two search arrays were generated for each parameter combination, resulting in a total of 256 stimuli. Since all stimuli were presented twice, the total number of trials was 512. For each search array, the noisy background was randomly selected from 10 noise images of 1000*1000 pixels. The final stimuli were resized to fully occupy the screen height during the online testing. Figure 5 shows an example of the stimuli used in Experiments 2a and 2b.

### Design & procedure

Participants were instructed to press ‘j’ if they found the target T and ‘f’ if they did not. The stimulus was present until response. After the initial response, participants could press the space bar within one second to reverse the response if they thought they made a motor error. Trial by trial feedback was not given, but after each block, the percentage correct was displayed. Experiment 2a generated 300 stimuli online for each participant. As in Experiment 1, the two copies of each of the 300 stimuli were randomly intermixed across six blocks of 100 trials. Experiment 2b used pre-generated stimuli as described in the Stimuli & Apparatus section for all participants. Two copies of the 256 pre-generated stimuli were randomly intermixed across four blocks of 128 trials. As in Experiment 1, this design had three factors, each with two levels: repetition, set size, and target. Participants were required to finish a 12-trial practice session before the experiment and would only be able to begin the experiment with an accuracy higher than 0.75, otherwise, they had to repeat the practice.

### Analysis method

The analysis of the RT data and miss rate data was the same as in Experiment 1. The RT data was subjected to a three-way repeated measure ANOVA with target presence, set size and repetition as within-subject factors. As in Experiment 1, there was no cueing intervention in Experiments 2a and 2b and therefore the same unique set of solution could be obtained for $d$, $s1$ and $s2$.

### Data exclusion

Experiment 2a required a screen resolution smaller than $2000\times 2000$ so that the noisy background would cover the whole screen. In Experiment 2a, one participant whose screen resolution did not meet the $2000\times 2000$ requirement was excluded. Next, the same exclusion criteria as in Experiment 1 were applied. Trials with 2.5 SD outlier RTs (3.33%) and motor correction (0.91%) were excluded for each observer. After removing both copies of the aforementioned trials, 93.52% remained. One participant was removed from Exp 2a based on a d’ of -0.04 (guessing). For the remaining participants, min d’ = 1.66, max d’ = 3.94.

For Experiment 2b, 2.80% and 0.39% of the trials were excluded due to outlier RTs and motor correction. 94.48% of the trials remained after removing both copies of those trials. No participant was removed based on the d’ calculated from the remaining trials (min d’ = 2.10, max d’ = 4.80).

## Results

### RTs

**Fig. 6:**
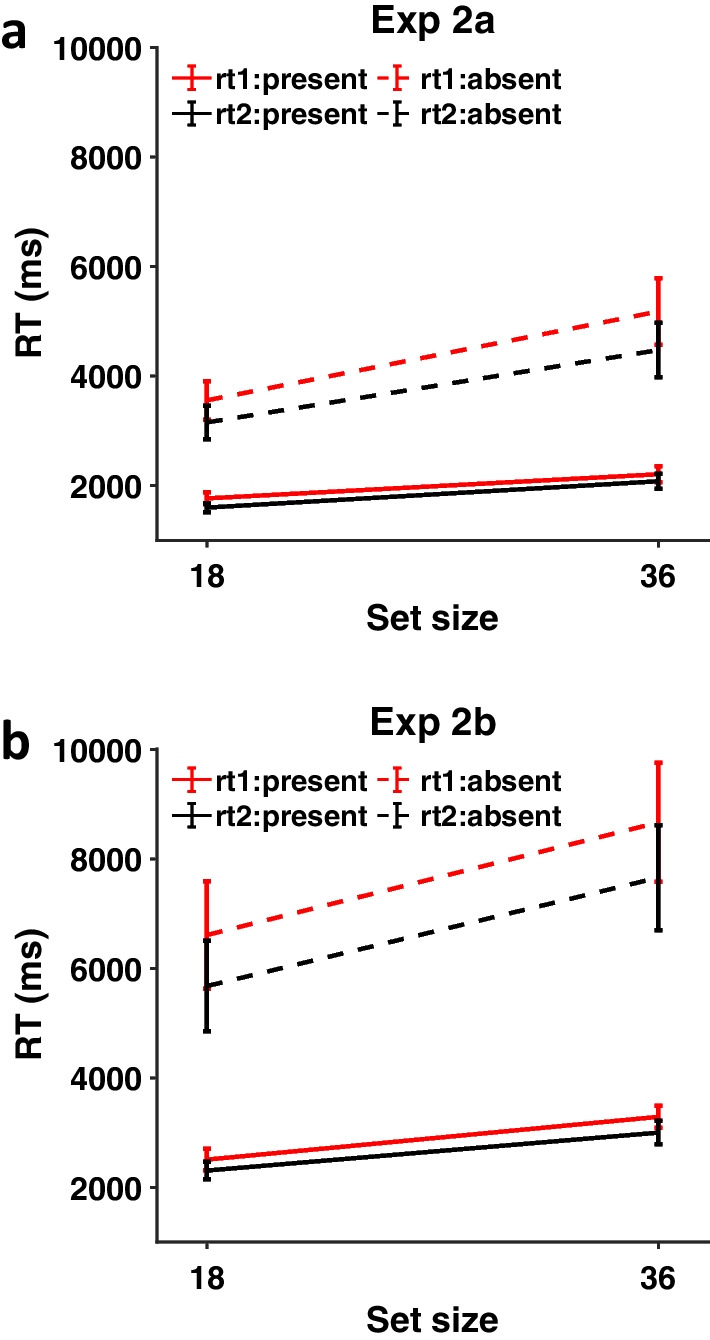
RTs from Experiments 2a and 2b as a function of set size, target presence, and repetition. Red lines: first presentation, Black lines: second presentation. Full lines: present trials, Dotted lines: absent trials. Error bars represent $\pm 1$ SEM

Figure 6 shows RTs on correct response trials from Experiments 2a and 2b. As can be seen, the second repetition of the trial is somewhat faster than the first, especially for absent trials. In Experiment 2a, the results from the three-way ANOVA suggest that there was a main effect of repetition [F(1, 18) = 11.84, *p* = 0.003, ${\eta }_{p}^{2}$ = 0.40]. The interaction between target presence and repetition [F(1, 18) = 4.80, *p* = 0.042, ${\eta }_{p}^{2}$ = 0.21] was also significant. Post hoc analyses suggest that the effect of repetition was significant on both target present [t(18) = 3.32, *p* = 0.004] and target absent trials [t(18) = 2.92, *p* = 0.009], but the effect of repetition was larger on target absent trials (Mean Difference = 554 ms) than on target present trials (Mean Difference = 148 ms). The interaction between set size and repetition was not significant [F(1, 18) = 3.49, *p* = 0.08, ${\eta }_{p}^{2}$ = 0.16]. The three-way interaction among all the factors was not significant either [F(1, 18) = 3.16, *p* = 0.09, ${\eta }_{p}^{2}$ = 0.15]. The full results of the three-way ANOVA are presented in Additional file 1: Figure S2. Individual RTs can be found in Additional file 1: Table S2.

In Experiment 2b, the results from the three-way ANOVA were similar. They show that there was a main effect of repetition [F(1, 20) = 18.06, *p* < 0.001, ${\eta }_{p}^{2}$ = 0.47]. The interaction between target presence and repetition was also significant [F(1, 20) = 9.78, *p* = 0.005, ${\eta }_{p}^{2}$ = 0.33]. Post hoc analyses found that the effect of repetition was significant on both target present [t(20) = 2.27, *p* = 0.034] and target absent trials [t(20) = 4.09, *p* < 0.001], but it was larger on target absent trials (Mean Difference = 970 ms) than on target present trials (Mean Difference = 224 ms). The interaction between set size and repetition was not significant [F(1, 20) = 0.48, p = 0.50, ${\eta }_{p}^{2}$ = 0.02]. The three-way interaction among all the factors was not significant either [F(1, 20) = 0.004, p = 0.95, ${\eta }_{p}^{2}$ = 0.00]. The full results of the three-way ANOVA are presented in Additional file 1: Figure S3 in the appendix. Individual RTs can be found in Additional file 1: Table S3.

### Miss rates

**Fig. 7:**
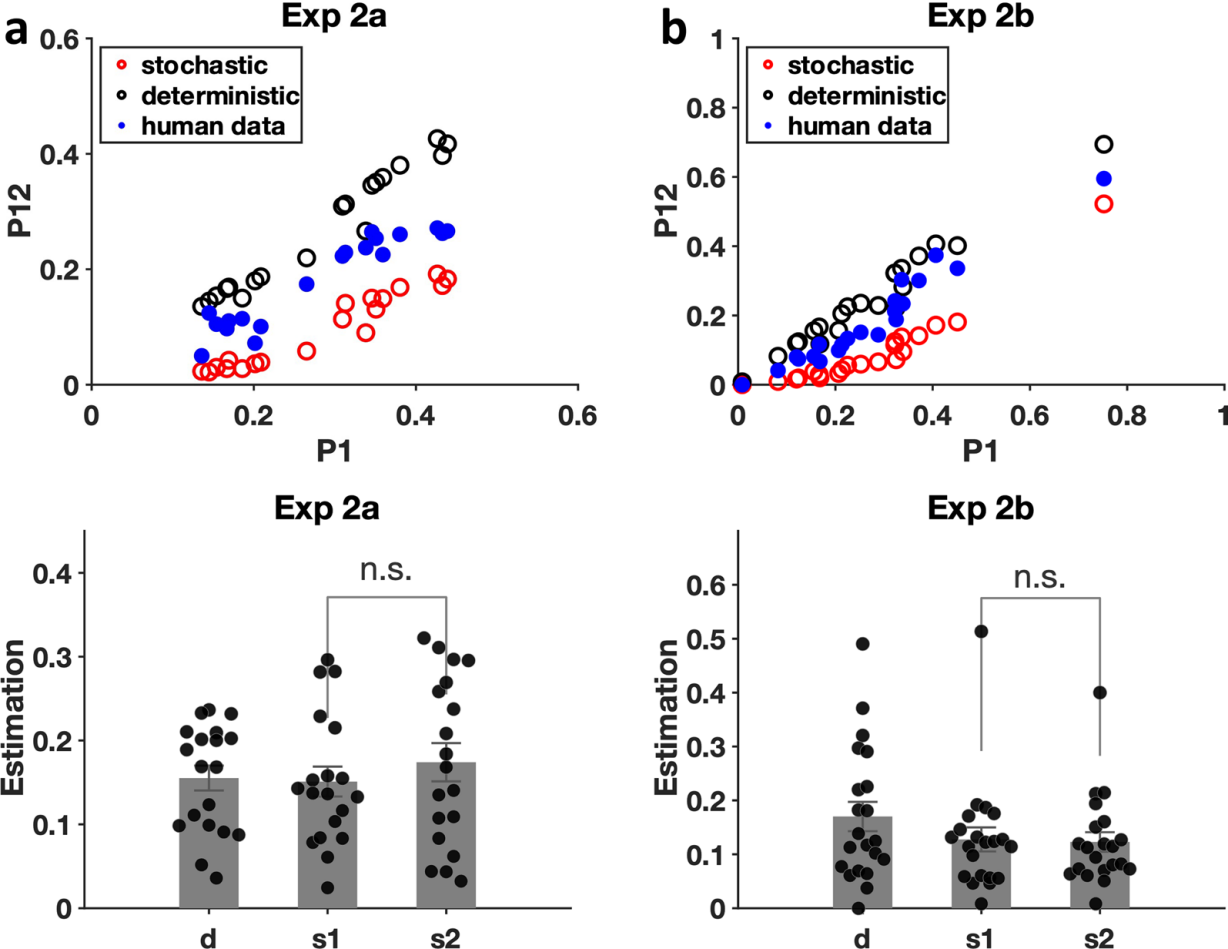
Miss rate analyses for Experiments 2a and 2b. **a** data from Experiment 2a. **b** data from Experiment 2b. Error bars represent $\pm 1$ SEM

As in Experiment 1, the miss errors are the main focus of interest here. Figure 7 shows the results of miss rate analyses for Experiments 2a and 2b. Compared to Experiment 1, it is clear that there were more errors and that those errors were less strictly stochastic. For Experiments 2a and 2b, the scatter plots show that the observed data (blue dots) lie between the deterministic (black circles) and the stochastic (red circles) predictions, indicating that the errors were a mix of both types. In Experiment 2a, a one-sample t-test showed that the deterministic error proportion $d$ was significantly different from 0 [t(18) = 10.45, *p* < 0.001, Cohen’s d = 2.40], suggesting the existence of deterministic errors in Experiment 2a. No learning effect was observed as suggested by the nonsignificant difference between $s1$ and $s2$ [t(18) = 1.52, *p* = 0.15, Cohen’s d = 0.35]. Experiment 2b essentially replicated the results from Experiment 2a. The deterministic error rate was significantly different from 0 [t(20) = 6.26, *p* < 0.001, Cohen’s d = 1.37]. The difference between $s1$ and $s2$ was not significant [t(20) = 0.34, *p* = 0.74, Cohen’s d = 0.07].

**Fig. 8:**
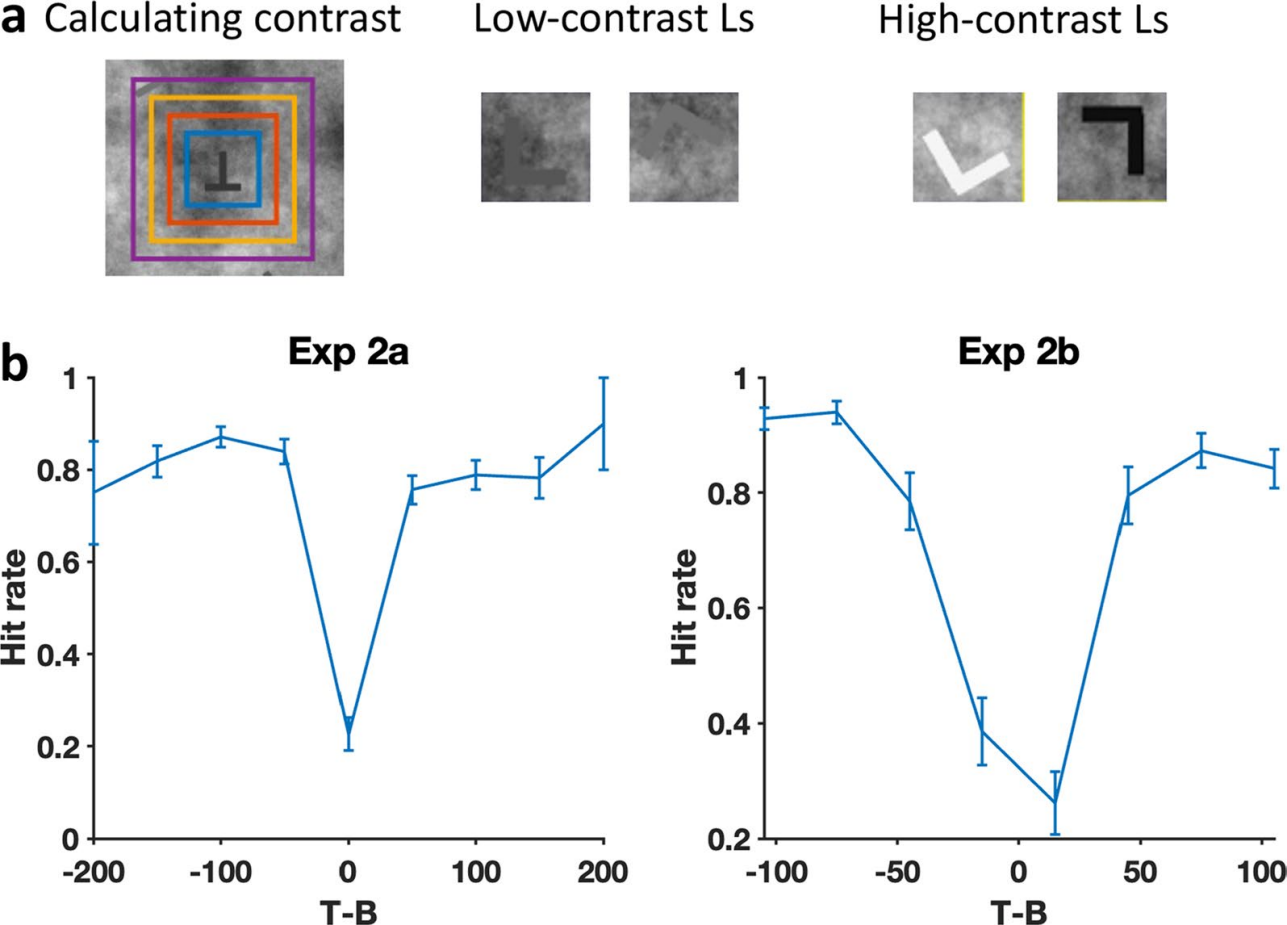
The effects of contrast on hit rate in Experiments 2a and 2b. **a** Example of contrast calculation. **b** Hit rate as a function of T-B. Data in Experiment 2a were binned (bin width: 50) to calculate the hit rate for each bin. Error bars represent $\pm 1$ SEM

As can be seen in Fig. 5, the letters in Experiments 2a and 2b were of varying contrast. Contrast on a non-uniform background can be defined in several ways (Peli, 1990). The precise details are not critical here. As cartooned in Fig. 8a, what matters is that low contrast items are harder to see and find than high contrast and, as shown in 8b, those low contrast images generate lower accuracy. For purposes of analysis, we computed the contrast as the target-background dissimilarity, i.e., target gray minus the average background gray in a square region surrounding the target. The exact size of the background region does not matter much based on the calculation results, so we chose the background patch outlined by the small blue square for the following analyses. The box's size is twice the length of the lines composing the letter T or L. As the “low contrast Ls” in Fig. 8 should illustrate, on a non-uniform background a letter may be detectable, even when T-B is near zero.

**Fig. 9:**
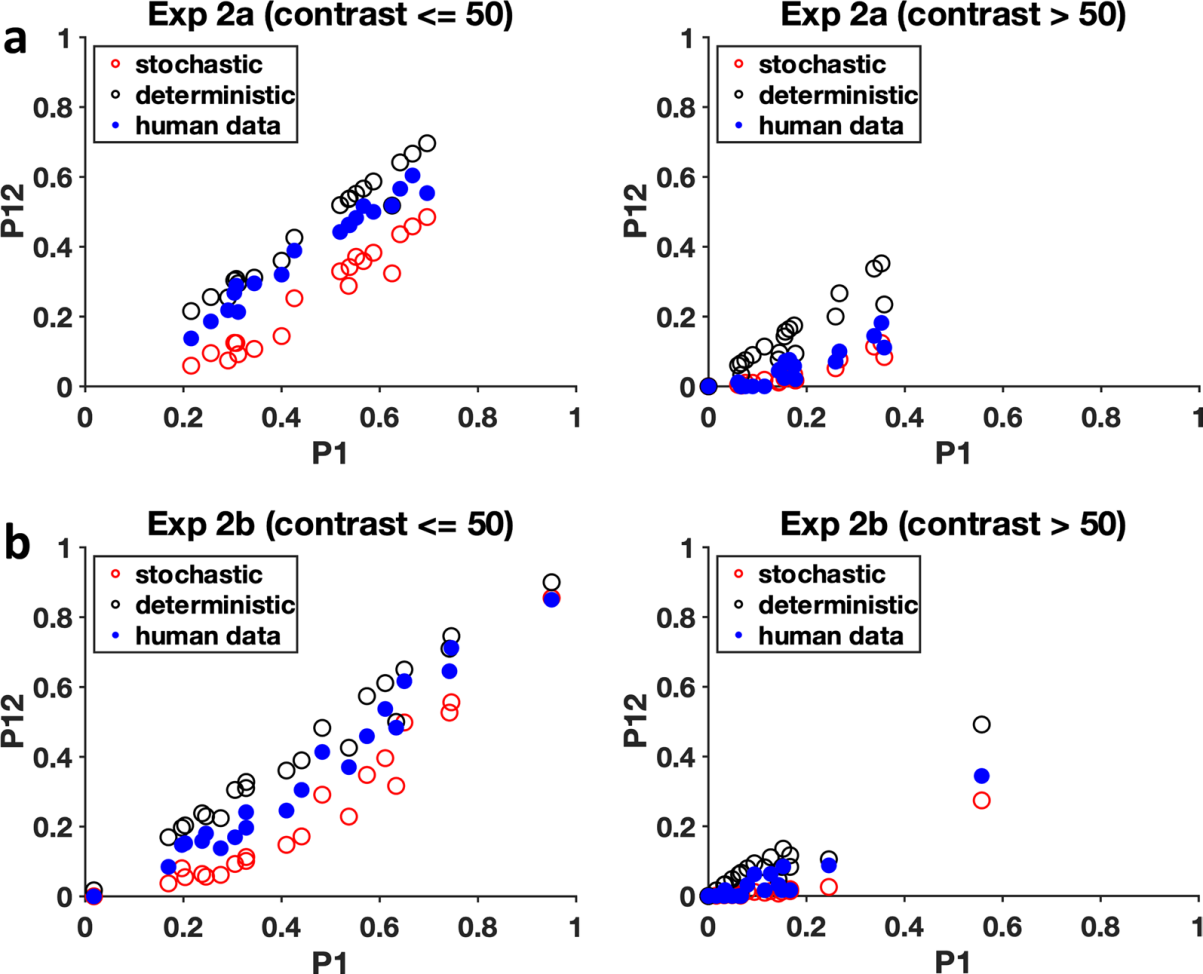
Error data for each observer (blue dots) for low contrast and higher contrast targets. Contrast was calculated by the absolute value of T – B. The red dots show the stochastic prediction and the black dots show the deterministic prediction

The impact of contrast on error rate is clearly illustrated in the graphs of Fig. 8b. Unsurprisingly, observers were far more likely to miss targets if those targets were of low contrast. Of more interest, if we separately analyse low contrast and higher contrast stimuli, we see that the low contrast errors are more likely to be deterministic while high contrast errors are largely stochastic. This is shown in Fig. 9.

### Experiment 2 discussion

Compared to Experiment 1, Experiments 2a and 2b involved a more difficult T-vs-L task where the target could be of very low contrast. RT data in Experiments 2a and 2b showed that observers still responded faster in round 2 than in round 1, as they did in Experiment 1. The smaller RT differences between round 1 and round 2 on target-present trials compared to those on target-absent trials are likely due to a floor effect since the task itself is a very simple visual search task with key press response. However, different patterns were observed in the miss rate data. Miss rate analyses demonstrated that while errors were purely stochastic in Experiment 1 they were a mix of deterministic and stochastic errors in the Experiment 2. When analysed separately, the low-contrast targets in Experiments 2a and 2b appeared to yield more deterministic errors. In addition, although there seemed to be some learning effect on RTs as in Experiment 1, no such effect was observed on search accuracy in these two experiments. It would not be terribly interesting to discover that observers do not find targets that they cannot see. However, in this case, these are targets that are *harder* to see, not impossible to see. The message of the two versions of Experiment 2 is that observers fairly reliably overlook some harder to see targets while also randomly missing some targets, whether they are hard to see or not.

What can be done to reduce these errors? It is possible that either the stochastic errors or the deterministic errors (or both) might be reduced if observers could be induced to pay attention to those items more effectively. Deployment of attention might be influenced in several ways. Two are tested in the following Experiment 3: 1) attention can be directed to regions of the display that might otherwise have been entirely overlooked; 2) attention can be directed to or enhanced at specific locations that might contain a target. Our goal here is test methods for directing attention that do not rely on knowledge about where the target actually resides or even where it is likely to reside. This is intended as generic advice to the searcher in contrast to the advice of an Artificial Intelligence system, trained to find specific target types.

## Experiments 3a, 3b and 3c: cueing interventions

In Experiments 3a, 3b and 3c, different forms of cueing interventions were introduced to direct attention in an effort reduce the errors. Experiments 3a and 3b moved attention around the field in an effort to decrease the chance of overlooking an item of interest. In Experiment 3a, the random cueing intervention was a yellow dot jumping to random places in the search display, summoning attention or the eyes to follow. In Experiment 3b, systematic area cueing was used. In this case, a transparent, outline square moved in a spiral path from center to periphery in the hope of inducing observers to search systematically and, perhaps, more exhaustively. Experiment 3c used item cueing. Each of the letters in the search display was highlighted by a yellow square around it in an effort to reduce the chance of missing a low contrast target. Highlighting each item is akin to having a system that figures out where all the interesting information might be but that cannot discriminate targets from distractors.

### Participants

Experiments 3a, 3b and 3c were run online on the Pavlovia platform (https://pavlovia.org). All the participants were recruited via Prolific. The estimated time of these three experiments was 1 h, based on which Prolific set the maximum time limit of 140 min. Participants who failed to finish the experiment within 140 min were labeled timed-out. The collected data included one timed-out participant in Experiment 3a and one in Experiment 3c. Time slots of timed-out participants were automatically released and were replaced by new ones on Prolific. Therefore, data from timed-out participants were not included in any of our analyses. In Experiment 3a, we tested 20 participants (6 males, 9 females, mean = 32.1, SD = 11.3, min = 22, max = 59, five participants did not provide their gender and age information). In Experiment 3b, we tested 20 participants (7 males, 11 females, mean = 27.9, SD = 6.2, min = 20, max = 40, two participants did not provide the gender and age information). In Experiment 3c, we tested 20 participants (9 males, 5 females, mean = 24.9, SD = 5.5, min = 19, max = 41, six participants did not provide the gender and age information). All participants reported normal or corrected-to-normal vision and gave their informed consent before they began the experiment. Participants received 8 GBP for their participation. Ethics approval came from The University of Manchester (2023–16117-29,230 for Exp 3a, 2023–16117-30,373 for Exp 3b, 2023–16117-30,584 for Exp 3c).

### Stimuli & apparatus

The exact same set of stimuli used in Experiment 2b were also used in Experiment 3a, 3b and 3c (Fig. 5b), but on the cued trials there was either a randomly moving yellow dot (Experiment 3a), an outline yellow square that moved in a spiral fashion (Experiment 3b), or a set of static yellow squares that highlighted the positions of all items (Experiment 3c).

### Design & procedure

**Fig. 10:**
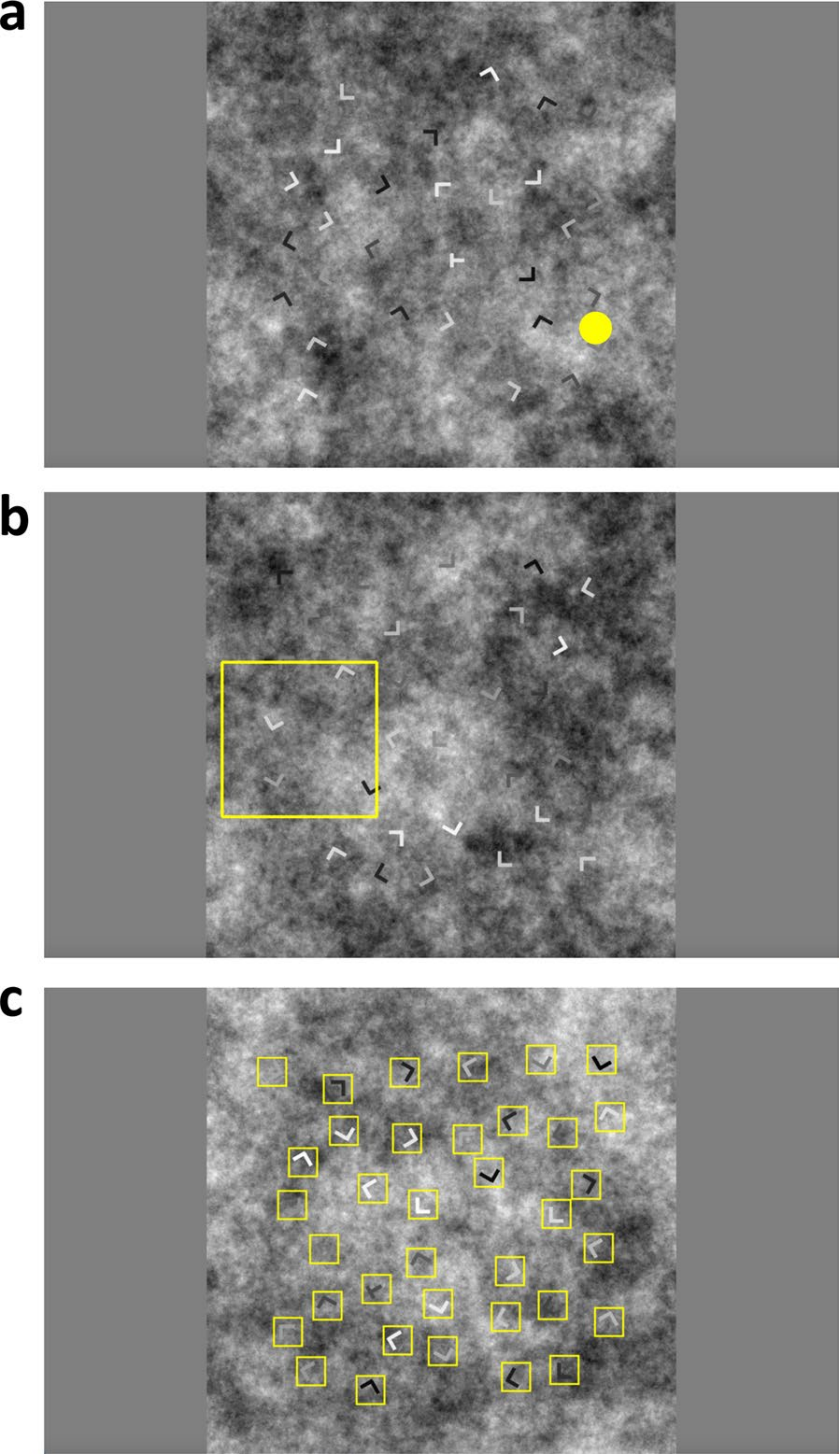
Illustration of the cueing intervention in Experiments 3a, 3b and 3c

Participants were instructed to press ‘j’ if they found the target T and ‘f’ if they did not. After the initial response, participants could press the space bar within one second to reverse the response if they thought that they made a motor error. The search time was limited to 20 s for Experiments 3a, 3b and 3c for the purpose of controlling the online experiment time. Trial by trial feedback was not given, but the percentage correct was displayed at the end of each block. The stimuli for Experiments 3a, 3b and 3c were the same as that of Experiment 2b, except for the introduction of the cueing intervention on half of the repetition trials. In Experiment 3a (Fig. 10a, the yellow dot is enlarged here for visualization), a yellow dot (size = 0.01 screen height) jumped at random places in the search display as the random cueing intervention, remaining at each location for 500 ms. In Experiment 3b (Fig. 10b), an outline square (size = 1/3 screen height) with yellow borders moved around the search display on systematic area cueing intervention trials, following a spiral path. Participants were instructed to follow the square when it appeared. In Experiment 3c (Fig. 10c), all the letters were highlighted by yellow squares around them (size = 0.06 screen height) as the item cueing intervention. In Experiments 3a and 3b, the cueing intervention was not related to the presence or the location of the target. In Experiment 3c, the presence of the cueing intervention was not related to the presence of the target either, but it did point out the positions of all the letters and thus also possibly the target. For half of the stimuli, we had no cue on the first copy of the stimulus but had a cue on the second copy (noCue – Cue condition). As comparison, for the other half of the stimuli, we had did not have a cue on the either the first or the second copy (noCue – noCue condition). All versions of Experiment 3 had therefore a design with four factors, each with two levels: repetition, cue, set size, and target. Participants were required to finish a 12-trial practice session before the experiment and would only be able to begin the experiment with an accuracy higher than 0.75, otherwise, they had to repeat the practice.

### Analysis method

We focused on the RT data and miss rate data with our primary interest in the error data as in the previous experiments. The four-way repeated measure ANOVA with target presence, set size, repetition and cueing intervention as within-subject factors was conducted for Experiments 3a, 3b and 3c. Since the three experiments here also involved a typical visual search task, it was expected to observe main effects of target presence (longer reaction times for absent trials), set size (longer reaction times for larger set sizes) and their two-way interaction (steeper reaction time slopes for absent trials). These effects will not be reported in detail in the following Results section. The results of the full ANOVA can be found in supplementary material.

To analyse the miss rate, we also calculated $P1$, $P2$ and $P12$ to estimate $d1$, $d2$, s1 and $s2$. For the noCue – noCue trials in Experiments 3a, 3b, 3c, there was no cueing intervention. A fixed deterministic rate was therefore assumed for round 1 and round 2, i.e., $d=d1=d2$. The solution of $d$, $s1$ and $s2$ was the same as in the previous experiments. For noCue – Cue trials in Experiments 3a, 3b and 3c, the cueing intervention was introduced on the second copy of trials. If the cueing intervention reduces deterministic errors, the assumption $d1=d2$ becomes $d1\ge d2$. This means that the assumption $d1=d2$ can no longer be used to arrive at a unique solution. $s2$ and $d2$ are still uniquely determined by solving the equations, but there are now multiple solutions for $s1$ and $d1$. However, considering that deterministic errors should be persistent when no additional intervention is implemented, the assumption $d1$ (noCue—Cue) = $d$ (noCue—noCue) should hold, thus leading to a unique solution for $s1$ in the noCue – Cue condition.

$$s2=\frac{P2-P12}{1-P1}$$

$$d2=\frac{P12-P1*P2}{1-P1-P2+P12}$$

$$s1=\frac{P1-d1}{1-d1}$$

It also should be noted that the split of stimuli into the noCue—Cue group and the noCue – noCue group was random for each participant. Therefore, it is possible that one group contained more deterministic error prone stimuli and the other group contained fewer such stimuli, but on average we should have $d1$ (noCue—Cue) = $d$ (noCue—noCue). If the noCue – noCue group contains more deterministic error prone stimuli than the other group, $d1$ (noCue – Cue) = $d$ (noCue – noCue) will lead to an overestimate of the actual $d1$ in the noCue – Cue group, which probably results in a negative $s1$ when the overestimate of $d1$ is larger than $P1$. To avoid such cases, any participant with a negative estimate of $s1$ was excluded when the analysis concerned the estimate of the deterministic error proportions $d1$/$d2$ and the stochastic error rates $s1$/$s2$.

### Data exclusion

The same exclusion criteria as in the previous experiments were applied for Experiments 3a, 3b and 3c. We removed trials with RTs smaller or greater than 2.5 SD from the mean RT in each condition for each observer (3.21% in Exp 3a, 3.22% in Exp 3b, 3.87% in Exp 3c). Then trials where participants corrected their motor responses were removed (0.70% in Exp 3a, 0.76% in Exp 3b, 0.69% in Exp 3c). When one trial was removed, the other copy of the trial was also removed (remaining trials: 93.77% in Exp 3a, 93.71% in Exp 3b, 92.42% in Exp 3c). After the removal of the above trials, we further checked the d’ of all the participants. Participants with d’ beyond 2.5 SD from the group mean for each individual experiment were excluded. One participant with d’ = 1.39 was removed from Exp 3a (for remaining participants, min d’ = 1.98, max d’ = 3.86). One participant with d’ = 0.69 was removed from Exp 3b (for remaining participants, min d' = 2.53, max d’ = 3.59). One participant with d’ = 1.68 was removed from Exp 3c (for remaining participants, min d’ = 2.38, max d’ = 4.11).

## Results

### RTs

**Fig. 11:**
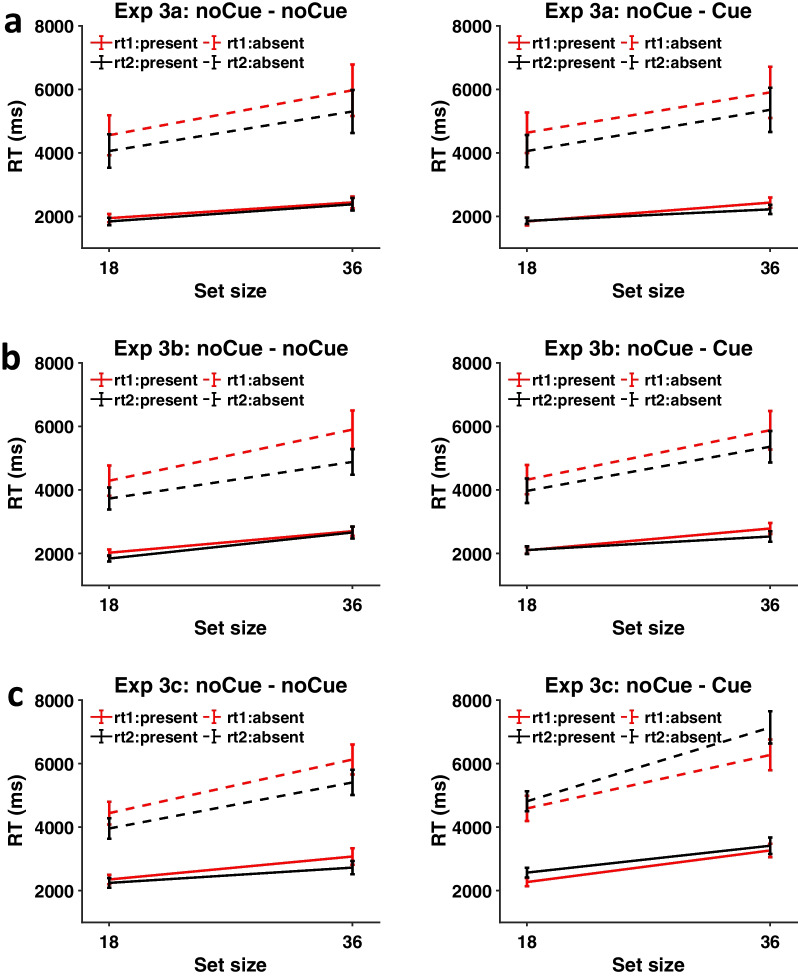
RTs from Experiments 3a, 3b and 3c as a function of set size, target presence, and repetition. Red lines: first presentation, Black lines: second presentation. Full lines: present trials, Dotted lines: absent trials. Error bars represent $\pm 1$ SEM

Figure 11 shows RTs on correct response trials from Experiments 3a, 3b and 3c. It appears that the cueing intervention had very little qualitative effect in Experiments 3a and 3b. RTs were faster on the second copy of the stimuli, especially for absent trials, but the presence or absence of the cueing intervention made little difference. In Experiment 3c, by contrast, the presence of the cue slowed the RT on the second appearance. Note that RT2 is faster when the cueing intervention is absent and slower when the cueing intervention is present. We can presume that the boxes marking all the items induced the observers to attend to more of the items or spend more time checking the highlighted areas.

To evaluate this statistically, four-way repeated measure ANOVAs with target presence, set size, repetition and cueing intervention as within-subject factors were conducted for Experiments 3a, 3b and 3c.

In Experiment 3a, the four-way interaction was significant [F(1, 18) = 6.61, p < 0.05, ${\eta }_{p}^{2}$ = 0.27], so two three-way ANOVAs with set size, repetition and cueing intervention as within-subjects factors were conducted for target present and target absent trials separately. On target present trials, the main effect of repetition was not significant [F(1, 18) = 3.25, *p* = 0.088, ${\eta }_{p}^{2}$ = 0.15], but on target absent trials, it was [F(1, 18) = 14.30, p = 0.001, ${\eta }_{p}^{2}$ = 0.44], showing that observers responded faster in round 2 than in round 1 for absent trials. The two-way interaction between repetition and cueing intervention was not significant for either target present [F(1, 18) = 0.028, *p* = 0.87, ${\eta }_{p}^{2}$ = 0.002] or target absent trials [F(1, 18) = 0.046, *p* = 0.83, ${\eta }_{p}^{2}$ = 0.003], suggesting that the cueing intervention did not have any effect on reaction time in Experiment 3a. The full results of the four-way ANOVA and three-way ANOVAs are presented in Additional file 1: Figures S4-1, S4-2 and S4-3. The individual RTs can be found in Additional file 1: Tables S4-1 and S4-2.

In Experiment 3b, the four-way interaction was also significant [F(1, 18) = 4.67, *p* < 0.044, ${\eta }_{p}^{2}$ = 0.21], so again two three-way ANOVAs were conducted for target present and target absent trials separately. On target present trials, the main effect of repetition was almost significant [F(1, 18) = 4.17, *p* = 0.056, ${\eta }_{p}^{2}$ = 0.19]. The two-way interaction between repetition and cueing intervention was not significant [F(1, 18) = 0.008, *p* = 0.93, ${\eta }_{p}^{2}$ = 0.00], suggesting that the cueing intervention did not influence RTs on target present trials. On target absent trials, the main effect of repetition was significant [F(1, 18) = 8.32, *p* = 0.01, ${\eta }_{p}^{2}$ = 0.32], showing that observers made faster responses in round 2 than in round 1 on target absent trials. The two-way interaction between repetition and cueing intervention was significant as well, [F(1, 18) = 4.95, *p* = 0.039, ${\eta }_{p}^{2}$ = 0.22]. The cueing intervention appears to have made observers search longer on target absent trials. The full results of the four-way ANOVA and three-way ANOVAs are presented in Additional file 1: Figures S5-1, S5-2 and S5-3. The individual RTs can be found in Additional file 1: Tables S5-1 and S5-2.

In Experiment 3c, the four-way interaction was again significant [F(1, 18) = 11.53, p = 0.003, ${\eta }_{p}^{2}$ = 0.39], so two three-way ANOVAs were conducted for target present and target absent trials separately. On target present trials, the main effect of repetition was not significant [F(1, 18) = 0.002, p = 0.96, ${\eta }_{p}^{2}$ = 0.00]. On target absent trials, the main effect of repetition was not significant either [F(1, 18) = 0.019, *p* = 0.89, ${\eta }_{p}^{2}$ = 0.001]. However, there was a strong two-way interaction between repetition and cueing intervention for both target present [F(1, 18) = 7.80, *p* = 0.012, ${\eta }_{p}^{2}$ = 0.30] and target absent trials [F(1, 18) = 45.79, *p *< 0.001, ${\eta }_{p}^{2}$ = 0.72], demonstrating that the cueing intervention in Experiment 3c slowed the search regardless of target presence. The effect of this item cueing intervention was larger on target absent trials (noCue – noCue: RT2 – RT1 = − 622 ms, noCue – Cue: RT2 – RT1 = 529 ms) than on target present trials (noCue – noCue: RT2 – RT1 = − 222 ms, noCue – Cue: RT2 – RT1 = 202 ms). The full results of the four-way ANOVA and three-way ANOVAs are presented in Additional file 1: Figures S6-1, S6-2 and S6-3. The individual RTs can be found in Additional file 1: Tables S6-1 and S6-2.

### Miss rates

**Fig. 12:**
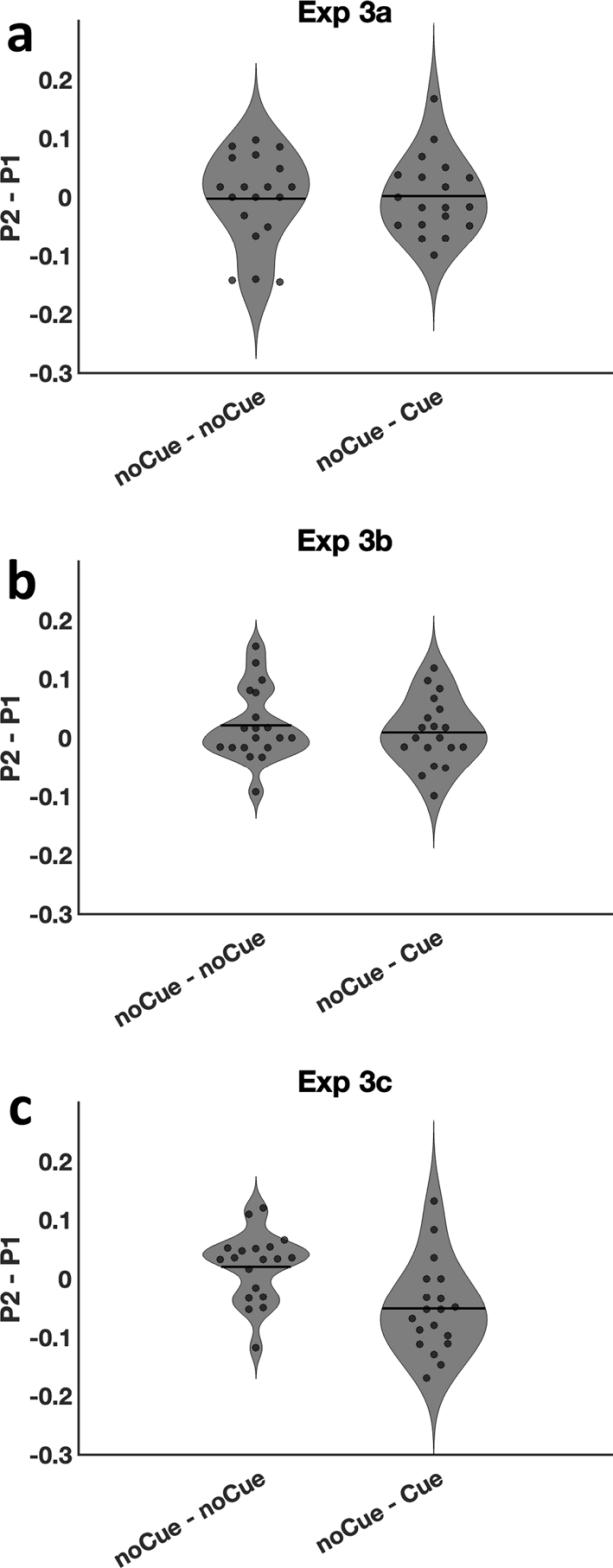
Miss rate difference (P2 – P1) for the noCue – noCue stimuli and noCue – Cue stimuli

Figure 12 shows the miss rate difference (P2–P1) for the noCue – noCue stimuli and the noCue – Cue stimuli. The critical comparison in all cases is between the miss rate on the second appearance compared to the first appearance, since the cueing intervention was only implemented on the second copy in the noCue – Cue group. Did the cueing intervention lower the miss error rate on the second appearance compared to when there was no cueing intervention? In Experiments 3a and 3b, the cueing intervention did not reduce the error rate [paired t-tests, 3a: t(18) = 0.19, *p* = 0.85, Cohen’s d = 0.04; 3b: t(18) = 0.63, *p* = 0.54, Cohen’s d = 0.14]. In Experiment 3c however, the drop in misses on second presentation was larger in the presence of the item cueing intervention than in the absence of the item cueing intervention [t(18) = 3.38, *p* = 0.0033, Cohen d = 0.78], suggesting that the cueing intervention in Experiment 3c effectively reduced errors.

**Fig. 13:**
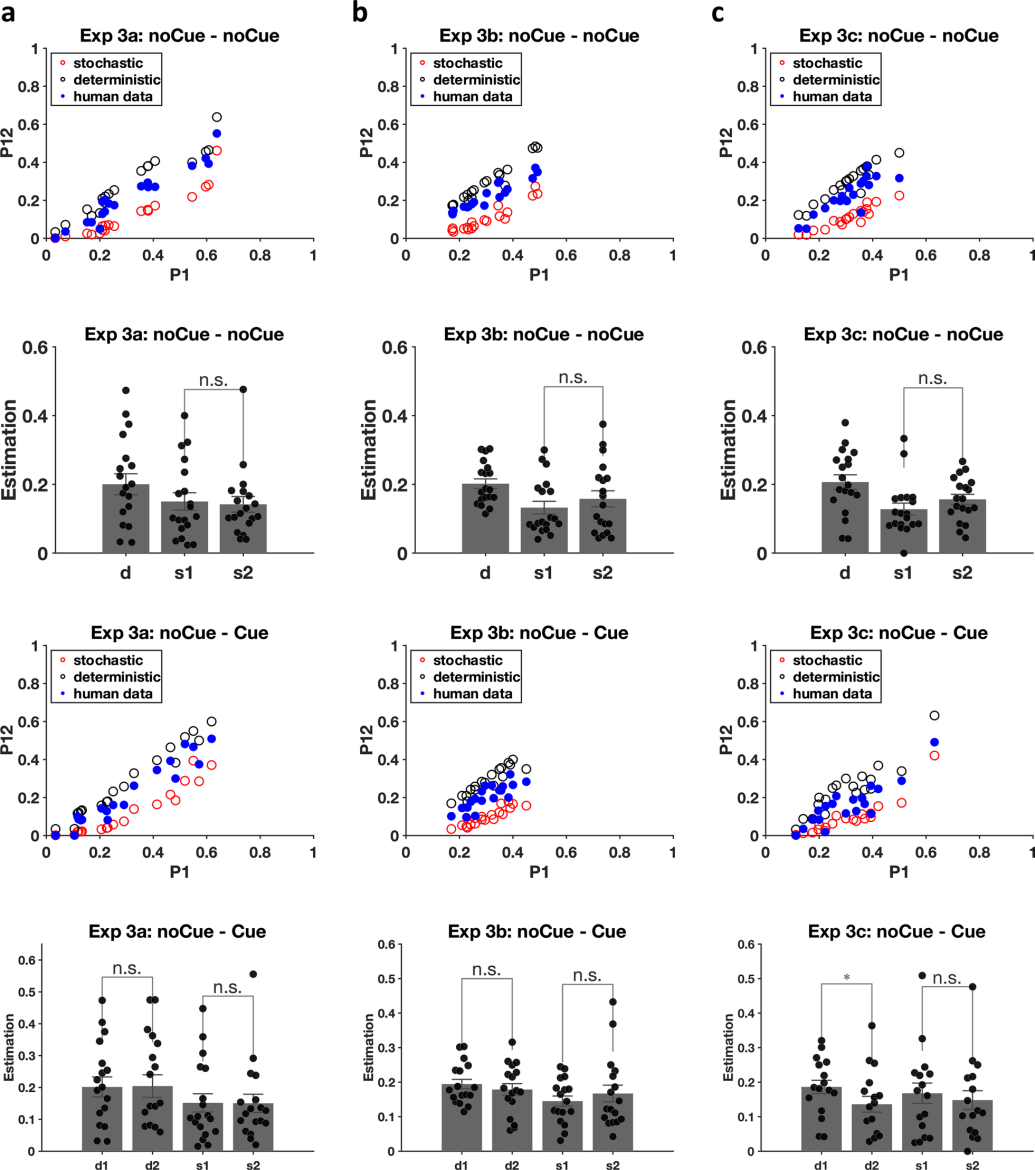
Miss rate analyses. **a** data from Experiment 3a. **b** data from Experiment 3b. **c** data from Experiment 3c. Error bars represent $\pm 1$ SEM

As can be seen in the scatter plots of Fig. 13, all versions of Experiment 3 replicated the main result of Experiment 2 in producing a mix of stochastic and deterministic errors: The observed data lie between the stochastic and deterministic predictions regardless of cue presence in Experiments 3a, 3b and 3c. As discussed in the *Analysis method* section, the proportion of deterministic errors and stochastic errors can be calculated by solving the relevant equations. For noCue – noCue trials in Experiments 3a, 3b, 3c, the proportion of deterministic errors was fixed for round 1 and round 2 as in the previous experiments ($d=d1=d2$), resulting in three parameters $d$, $s1$ and $s2$. For the noCue – Cue trials in Experiments 3a, 3b, 3c, the presence of the cueing intervention could influence the proportion of either deterministic or stochastic errors (or both) in round 2. Thus there were four parameters $d1$, $d2$, $s1$ and $s2$. Unique solutions could still be obtained for $d2$ and $s2$ by solving the equations, but there could be multiple solutions for $d1$ and $s1$. Under the assumption that the deterministic errors should be persistent when no interference was added, the $d1$ parameter in the noCue – Cue condition was taken to be identical with $d$ from the noCue – noCue condition. This could be used to derive a unique solution for $s1$.

In Experiment 3a, the noCue – noCue trials replicated the results from Experiments 2a and 2b. The deterministic error rate was significantly different from 0 [t(18) = 6.56, *p* < 0.001, Cohen’s d = 1.51] and no learning effect was found [t(18) = 0.32, *p* = 0.76, Cohen’s d = 0.07]. For the noCue – Cue trials, the critical comparisons are between d1 and d2 and/or s1 and s2. Did the random cueing intervention reduce the error rate? One participant got a negative $s1$ after we replaced $d1$ (noCue—Cue) with $d$ (noCue—noCue) and was therefore excluded from the following analysis. Neither the deterministic proportions nor the stochastic rates were significantly different between round 1 and round 2 [$d1$ vs. $d2$: t(17) = 0.18, *p* = 0.86, Cohen’s d = 0.04; $s1$ vs. $s2$: t(17) = 0.04, *p* = 0.97, Cohen’s d = 0.01], suggesting that the random cueing intervention in Experiment 3a failed to reduce either type of errors.

Results from Experiment 3b were similar to the results from Experiment 3a. For the noCue – noCue trials, the deterministic error rate was significantly different from 0 [t(18) = 14.39, *p* < 0.001, Cohen’s d = 3.30] and no learning effect was found [t(18) = 1.42, *p* = 0.17, Cohen’s d = 0.33]. For the noCue – Cue trials, two participants were excluded due to negative $s1$. The systematic area cueing intervention also failed to reduce either type of errors [$d1$ vs. $d2$: t(16) = 0.80, *p* = 0.43, Cohen’s d = 0.19; $s1$ vs. $s2$: t(16) = 0.72, *p *= 0.48, Cohen’s d = 0.17].

In Experiment 3c, the results for the noCue – noCue trials were, again, the same as in the previous experiments. The deterministic error rate was significantly different from 0 [t(18) = 9.76, *p* < 0.001, Cohen’s d = 2.24] and no learning effect was found [t(18) = 1.64, *p* = 0.12, Cohen’s d = 0.38]. However, the noCue – Cue trials produced a different, more interesting result in Experiment 3c. Three participants were excluded due to negative $s1$. Importantly, the comparison between $d1$ and $d2$ for the remaining participants did show a significant difference. $d2$ was smaller than $d1$ [t(15) = 2.69, *p* = 0.017, Cohen’s d = 0.67], suggesting that the item cueing intervention in Experiment 3c effectively reduced deterministic errors. No significant effect was found on stochastic errors [t(15) = 0.86, *p* = 0.40, Cohen’s d = 0.22].

### Experiment 3 discussion

Experiments 3a, 3b and 3c used the same set of stimuli as in Experiment 2b but introduced cueing interventions in an attempt to reduce error rates. Experiments 3a and 3b were efforts to spread attention around the display without needing to know anything about the contents of the display. In Experiment 3a, this was implemented as a dot that jumped to random locations. In Experiment 3b, an outline square moved systematically. Neither of these interventions had an impact on the errors.

However, the item cueing intervention in Experiment 3c did have an effect. In Experiment 3c, when all of the locations of items were outlined on the screen, participants slowed down, compared to the noCue—noCue condition. More importantly, miss error rates were reduced. The analysis of those errors indicates that the intervention in Experiment 3c had its biggest effect on deterministic errors. It seems likely that the outline boxes directed attention to some lower contrast items that might have otherwise been overlooked. Paired t-tests suggest that for the noCue – Cue stimuli in Experiment 3c, the target contrast in correct response trials was significantly lower in round 2 than in round 1[t(18) = 6.13, *p* < 0.001, Cohen’s d = 1.41] while in all other situations the difference was not significant.

## General discussion

Search errors are ubiquitous in tasks from the lab and real-life. Although it is unlikely that such errors could ever be completely eliminated (Brady, 2017), efforts to reduce errors are still worthwhile and hold significant potential to improve performance on socially important search tasks. In this paper, we were interested in the nature of miss errors in a simple laboratory-based search task. We chose a typical T-vs-L task but, we presume, that choice is not critical. Even in such a simple task, errors still occur at a steady rate. Those errors could be purely stochastic, purely deterministic or a mix of both types of errors. Our approach to distinguishing stochastic from deterministic contributions to errors was to show each display twice in the experiment. The straight-forward logic is that a stochastic error, made on one appearance of a display, tells you nothing about whether it will be missed on the second appearance of the displays. On the other hand, if that target is missed for some entirely deterministic cause, it would definitely be missed again at the next opportunity. Six experiments with repeated displays were conducted. In Experiment 1, all the letters were white and presented against a uniform gray background. The target letter was always clearly visible when present in the search array. Our analysis showed that the errors in this experiment were almost purely stochastic. In Experiments 2a and 2b, the letters were of different grayscale values and were presented against a noisy background. The target letters varied from clearly visible to low contrast. Our results suggested that the errors in Experiments 2a and 2b were a mix of both types of errors with lower contrast targets accounting for more of the deterministic errors. Experiments 3a, 3b and 3c used the same stimuli as in Experiment 2 and attempted to reduce the errors with different forms of cueing interventions. In Experiment 3a, a yellow dot jumped at random places in the search display on some trials, remaining at each location for 500 ms. This was to enhance observers’ attention at those locations. In Experiment 3b, an outline square with yellow borders appeared on some trials, following a spiral path. This intervention was intended to guide observers to search the entire display. In Experiment 3c, all the letters were highlighted by yellow squares around them on the cued trials. Our results suggest that only the item cueing intervention that had knowledge of item locations could effectively reduce the errors and the reduced errors were mainly deterministic errors. These results make it less likely that an intervention that is truly agnostic about the search display would be helpful. That said, one could try more forceful efforts to get participants to look at “everything” and, thus, not overlook targets like low contrast items. For instance, military surveillance officers used to divide large aerial photographs into a grid of smaller regions and systematically mark each region to indicate that it had been examined. This could reduce errors caused by simply overlooking some region. Of course, such a protocol greatly increases the time per image. In 3a and 3b, we attempted to get a similar benefit at less of a cost. Sadly, we did not succeed. In situations where it is worth paying the cost in time, a more mandatory style of intervention could be tried.

When it comes to some real-life tasks, the target might not be as specific as the letter T in our task. The target definition might be broader (e.g. find animals) and/or the target might be more ambiguous (Is that really a cancerous skin lesion?). There have been other attempts to reduce the errors in these more complex situations. For example, Nartker et al. (2020) tested three different methods to reduce categorical errors in a “mixed hybrid search” task (Wolfe et al., 2017) where participants searched for a list of targets; some specific (this hammer) and some categorical (any animal). In mixed hybrid search, participants tended to miss more categorical targets. To reduce such errors, several strategies were tried: (1) boosting categorical targets in memory; (2) separating the responses for specific and categorical targets; (3) full check list procedure that required participants to make an explicit response to the presence or absence of each type of target. Of all these measures, only the full checklist procedure effectively reduced categorical target errors. As with dividing an aerial image into little squares, this improvement comes at the expense of substantially longer reaction time.

Low-prevalence targets are also more frequently missed. Horowitz (2017) summarized some of the experimental manipulations that have be tried to reduce those errors. These include introducing a regime of brief retraining periods with high prevalence and full feedback (Wolfe et al., 2007), reducing the uncertainty of examined area by eye movement feedback (Drew & Williams, 2017; Peltier et al., 2017a, 2017b) and providing an opportunity to correct motor errors (Fleck & Mitroff, 2007). The success of such methods is mixed. Of note, our random cueing intervention in Experiment 3a and systematic area cueing intervention in Experiment 3b both attempted to move attention around so as to decrease the chance of overlooking certain areas, the idea of which was similar to the eye movement feedback method proposed by Drew et al. (2017) and Peltier et al. (2017). However, all the methods in this vein have failed to effectively reduce search errors. In contrast to the manipulations of the task, other researchers have focused more on individual differences to identify those who are likely to perform better on a low prevalence search task (Peltier & Becker, 2017a, 2017b, 2020). Such individual approaches could also provide some insights about how to improve real-world visual search performance.

In summary, errors in visual search are ubiquitous and stubborn. Our results suggest that errors may be almost completely stochastic when targets are clearly visible. Such errors may be hard to be reduced by any method that does not come down to spending more time and “paying” more attention. When targets are harder, but not impossible, to see, more of those hard to see targets appear to be missed in a deterministic manner. In the present experiments, deterministic errors due to low target contrast were reduced to some extent by an appropriate, rather simple intervention. Drawing attention to targets that might otherwise be reliably overlooked seems like a potentially promising approach to reducing LBFTS errors.

### Supplementary Information

**Additional file1**. Detailed Model Illustration and RT analyses.

## Data Availability

Experiments 2a, 3b, 3a, 3b, 3c were preregistered on OSF. Experiment 2a: https://osf.io/c6usk Experiment 2b: https://osf.io/z9mb3 Experiment 3a: https://osf.io/nfpwc Experiment 3b: https://osf.io/dmh7s Experiment 3c: https://osf.io/83f2r Data and code can be found at https://osf.io/c3yjz/.

## Footnotes

1 One of our reviewers (Michael C. Hout) pointed out that this model ignores the possibility of “lucky hits”, where a participant gives a present response even though they have not found the target. This can happen both for fundamentally findable targets and fundamentally unfindable targets. A full model that incorporates lucky hits is presented in Additional file 1: Appendix B. We used the false alarms rates in round 1 and round 2 to estimate the lucky hit rates because the false alarm rates give an indication of how often participants give a present response without finding the target. The simplified model instead of the full model is adopted in the remainder of this paper, because the low false alarm rates in all our experiments (the average is 1%) indicate that the rate of lucky hits would not have a significant impact on the results.
