# Supplementary material for: Errors in visual search: Are they stochastic or deterministic?
Source: Cogn Res Princ Implic. 2024 Mar 19;9:15. doi: 10.1186/s41235-024-00543-z (PMC10951178; doi:10.1186/s41235-024-00543-z)
Supplement: Supplementary file 1 — Additional file1. Detailed Model Illustration and RT analyses. [file 41235_2024_543_MOESM1_ESM.docx]

## Appendix A: Illustration of the simplified model


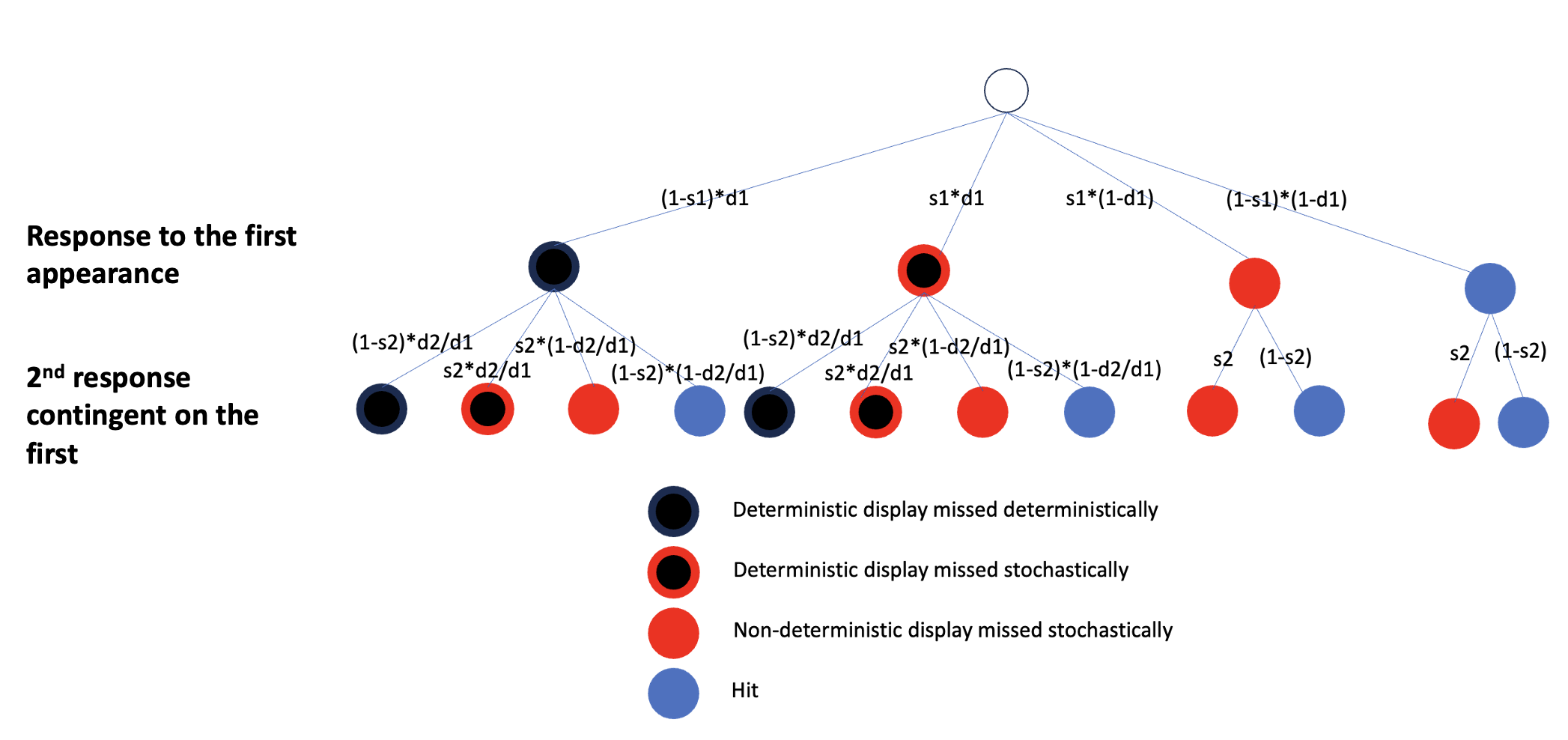


- $P1$ is the sum of the probability of the black circle, the black circle with the red border and the red circle in row 1.

$$P1=\left( 1-s1 \right)*d1+s1*d1+s1*\left( 1-d1 \right)=\left( 1-s1 \right)*d1+s1=d1-s1*d1+s1=d1*\left( 1-s1 \right)+s1$$

- $P2$ is the sum of probability of the black circles, the black circles with the red border and the red circles in row 2.

$$P2=\left[ \left( 1-s1 \right)*d1+s1*d1 \right]*\left[ \left( 1-s2 \right)*\frac{d2}{d1}+s2*\frac{d2}{d1}+s2*\left( 1-\frac{d2}{d1} \right) \right]+s1*\left( 1-d1 \right)*s2+\left( 1-s1 \right)*\left( 1-d1 \right)*s2=d1*\left( \frac{d2}{d1}+s2-s2*\frac{d2}{d1} \right)+\left( 1-d1 \right)*s2=d2+s2*d1-s2*d2+\left( 1-d1 \right)*s2=d2+s2*\left( d1-d2 \right)+\left( 1-d1 \right)*s2$$

- $P12$ is the sum of probability of the black circles, the black circles with the red border and the red circles in row 2 excluding the last red circle stemmed from a hit in row 1.

$$P12=\left[ \left( 1-s1 \right)*d1+s1*d1 \right]*\left[ \left( 1-s2 \right)*\frac{d2}{d1}+s2*\frac{d2}{d1}+s2*\left( 1-\frac{d2}{d1} \right) \right]+s1*\left( 1-d1 \right)*s2=d2+s2*\left( d1-d2 \right)+s1*\left( 1-d1 \right)*s2$$

## Appendix B: Full model considering lucky hit

*
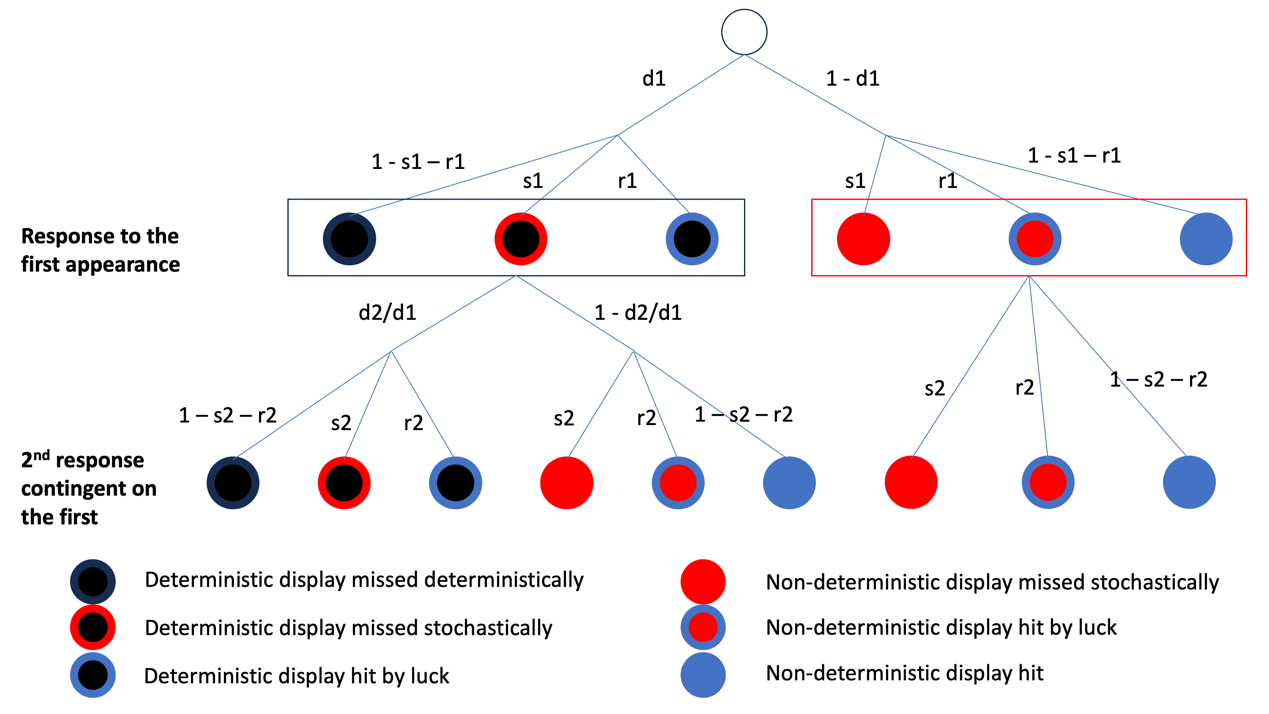
*

In the full model, there are six parameters: $d1$ and $d2$ for the deterministic rates in round 1 and round 2, $s1$ and $s2$ for the stochastic rates in round 1 and round 2, and $r1$ and $r2$ for the lucky hit rates in round 1 and round 2.

The proportion besides each line is relative to the element that leads to the split of lines (for each split of lines, the sum of probability will always be 1).

- $P1$ is the sum of the probability of errors in row 1.

$$P1=\left( 1-r1 \right)*d1+s1*\left( 1-d1 \right)$$

- $P2$ is the sum of probability of errors in row 2.

$$P2=d2*(1-r2)+s2*\left( d1-d2 \right)+\left( 1-d1 \right)*s2$$

- $P12$ is the sum of is the sum of probability of errors in both row 1 and row 2.

$$P12=\left( 1-r1 \right)*\left( 1-r2 \right)*d2+s2*\left( 1-r1 \right)*\left( d1-d2 \right)+s1*\left( 1-d1 \right)*s2$$

After some straightforward but rather tedious mathematics we can derive the following expressions for d1, d2, s1, and s2:

$$d2=(P12-P1*P2))/((r2-1)*r1- P2+P12+(1-P1)*(1-r2)+ P2*r1)$$

$$s2=(P2-d2*(1-r2))/(1-d2)$$

$$s1=(P1-d1*(1-r1))/(1-d1)$$

$$d1=(P1-s1)/(1-s1-r1)$$

The false alarm rate in round 1 and round 2 can be used to estimate the values of $r1$ and $r2$. The false alarm rate gives an indication for the propensity of participants to give a present response without finding the target.

If we assume that $r1=r2=0$, these expressions become:

$$d2=(P12-P1*P2))/(1-P1-P2+P12)$$

$$s2=(P2-d2)/(1-d2)$$

$$s1=(P1-d1)/(1-d1)$$

$$d1=(P1-s1)/(1-s1)$$

If we further assume that $d1=d2=d$, these expressions revert to the ones derived for the simplified model that is used throughout the paper

$$d=(P12-P1*P2))/(1-P1-P2+P12)$$

$$s1=(P1-P12)/(1-P2)$$

$$s2=(P2-P12)/(1-P1)$$

## Appendix C: Analyses on RTs

**Table S1. RTs (ms) in Experiment 1.**

| Target present | | | | Target absent | | | |
| --- | --- | --- | --- | --- | --- | --- | --- |
| Ss = 18 | | Ss = 36 | | Ss = 18 | | Ss = 36 | |
| Rep1 | Rep2 | Rep1 | Rep2 | Rep1 | Rep2 | Rep1 | Rep2 |
| 961 | 991 | 1336 | 1248 | 1513 | 1531 | 2890 | 2458 |
| 1063 | 1025 | 1409 | 1371 | 1919 | 1981 | 2993 | 3167 |
| 1189 | 1123 | 1865 | 1465 | 2683 | 2708 | 4423 | 4013 |
| 883 | 735 | 905 | 867 | 1060 | 935 | 1296 | 1117 |
| 1139 | 977 | 1200 | 1057 | 1910 | 1843 | 2593 | 2622 |
| 1125 | 961 | 1530 | 1145 | 2055 | 1479 | 2718 | 2303 |
| 1101 | 954 | 1562 | 1339 | 2751 | 2380 | 3941 | 3040 |
| 1117 | 823 | 1511 | 1175 | 1979 | 1657 | 2714 | 2251 |
| 1392 | 1181 | 1627 | 1360 | 2732 | 2771 | 3899 | 4242 |
| 805 | 727 | 1186 | 1132 | 1749 | 1888 | 2653 | 2922 |
| 882 | 803 | 983 | 966 | 1571 | 1838 | 2269 | 2545 |
| 912 | 837 | 1298 | 1211 | 1984 | 1678 | 3307 | 2803 |
| 1306 | 1273 | 1376 | 1442 | 2231 | 2285 | 2922 | 4159 |
| 1163 | 1041 | 1723 | 1517 | 2443 | 2556 | 3609 | 3413 |
| 937 | 863 | 1154 | 997 | 2126 | 1746 | 3186 | 2649 |
| 837 | 729 | 979 | 866 | 1681 | 1753 | 2522 | 2323 |
| 1095 | 1019 | 1399 | 1171 | 2692 | 2254 | 3440 | 3154 |
| 1515 | 1320 | 2040 | 1903 | 2844 | 2993 | 4351 | 4654 |
| 969 | 820 | 1108 | 1048 | 2400 | 2575 | 3866 | 3666 |


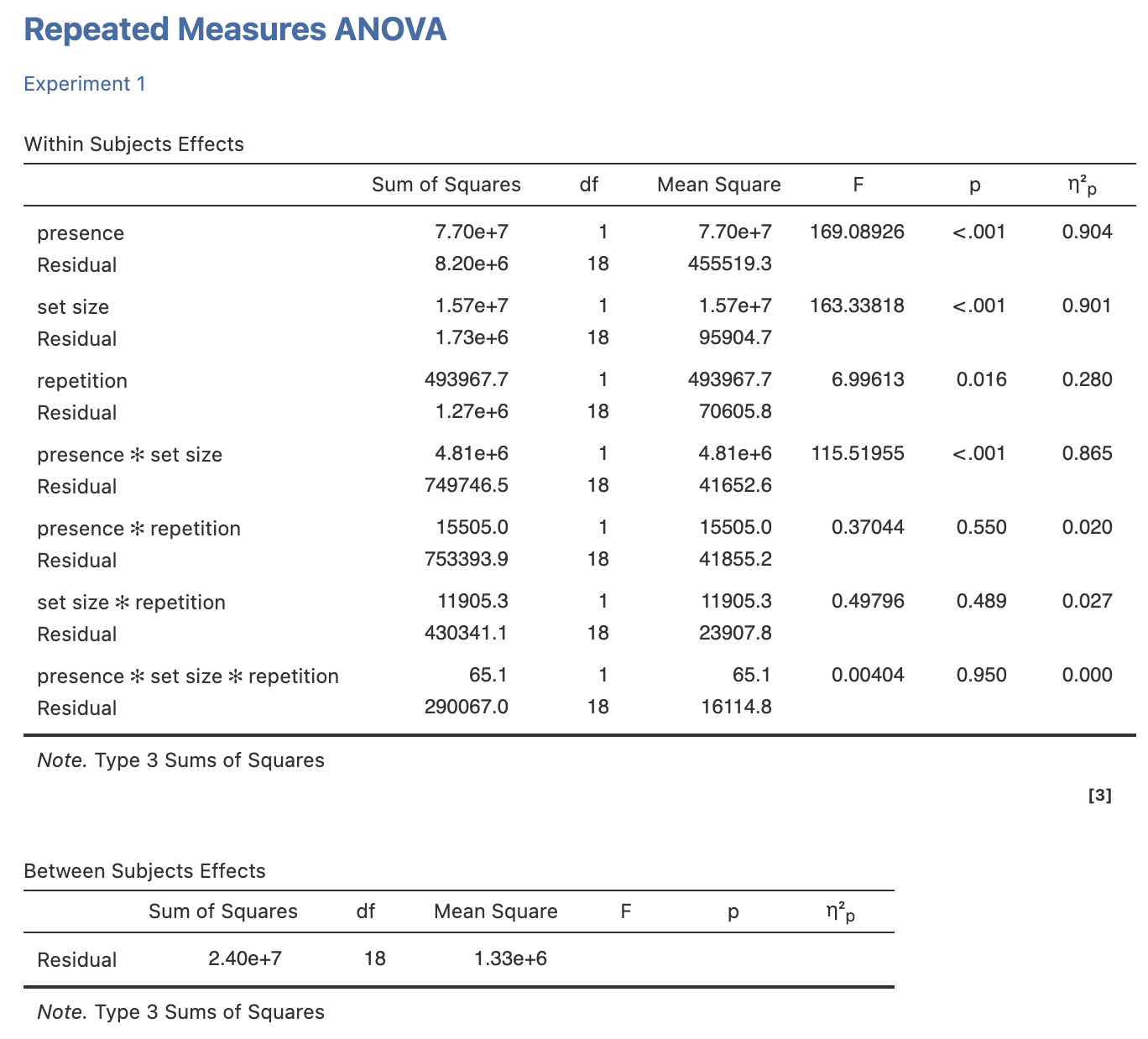


**Figure S1. RT analyses for Experiment 1.**

**Table S2. RTs (ms) in Experiment 2a.**

| Target present | | | | Target absent | | | |
| --- | --- | --- | --- | --- | --- | --- | --- |
| Ss = 18 | | Ss = 36 | | Ss = 18 | | Ss = 36 | |
| Rep1 | Rep2 | Rep1 | Rep2 | Rep1 | Rep2 | Rep1 | Rep2 |
| 1354 | 1401 | 1383 | 1361 | 2005 | 1526 | 2674 | 2169 |
| 1345 | 1215 | 1674 | 1419 | 2107 | 1863 | 2586 | 2373 |
| 2034 | 1750 | 1771 | 2005 | 2224 | 2228 | 3052 | 2746 |
| 1469 | 1248 | 1926 | 1535 | 3934 | 2348 | 5428 | 3211 |
| 1929 | 1651 | 2385 | 2208 | 4367 | 4082 | 6865 | 6749 |
| 1278 | 1299 | 1527 | 1466 | 1912 | 1877 | 2493 | 2707 |
| 1426 | 1454 | 1661 | 1664 | 2255 | 1964 | 3066 | 2524 |
| 2071 | 1807 | 3254 | 2603 | 5957 | 5653 | 8974 | 9352 |
| 1381 | 1397 | 1638 | 1706 | 2639 | 2320 | 3550 | 3350 |
| 2508 | 2341 | 3421 | 3260 | 5584 | 4415 | 8450 | 6582 |
| 1435 | 1360 | 1837 | 1866 | 2396 | 2255 | 2886 | 2923 |
| 2075 | 1698 | 2176 | 2438 | 3996 | 3477 | 6230 | 3981 |
| 1550 | 1379 | 2222 | 2041 | 3772 | 3657 | 5559 | 4976 |
| 1667 | 1576 | 1921 | 2228 | 2594 | 3027 | 4106 | 4157 |
| 1291 | 1329 | 1839 | 1674 | 2409 | 2171 | 3300 | 2812 |
| 1737 | 1230 | 2400 | 1595 | 3065 | 2216 | 3686 | 3095 |
| 2515 | 2213 | 3555 | 3339 | 7738 | 5897 | 13063 | 9011 |
| 1444 | 1487 | 2121 | 2062 | 3268 | 3207 | 5119 | 4611 |
| 3030 | 2508 | 3200 | 3015 | 5343 | 5728 | 7322 | 7662 |


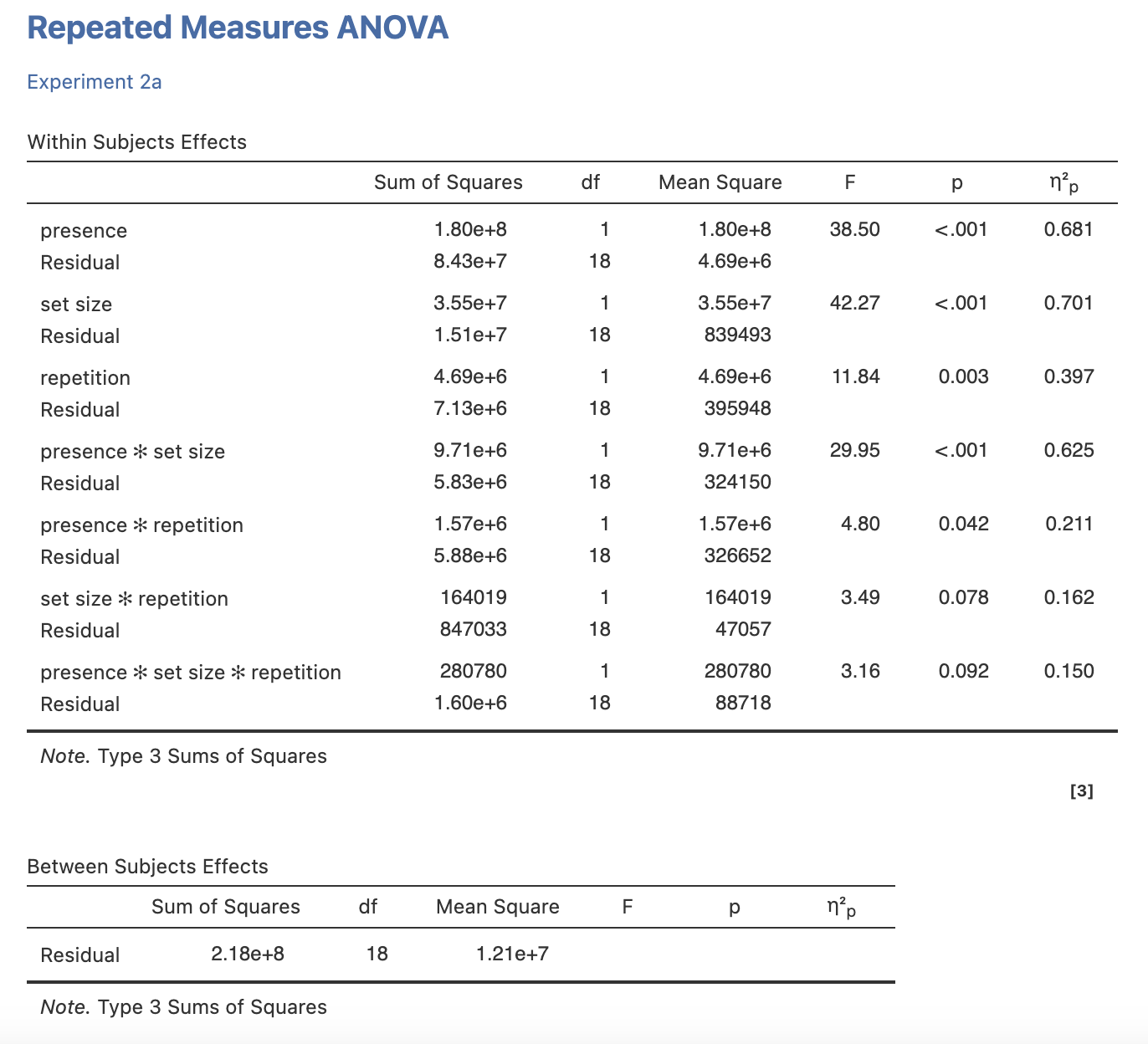


**Figure S2. RT analyses for Experiment 2a.**

**Table S3. RTs (ms) in Experiment 2b.**

| Target present | | | | Target absent | | | |
| --- | --- | --- | --- | --- | --- | --- | --- |
| Ss = 18 | | Ss = 36 | | Ss = 18 | | Ss = 36 | |
| Rep1 | Rep2 | Rep1 | Rep2 | Rep1 | Rep2 | Rep1 | Rep2 |
| 2370 | 2393 | 2342 | 2423 | 3047 | 2888 | 3716 | 3766 |
| 1879 | 2074 | 2912 | 2416 | 3933 | 3038 | 6648 | 4758 |
| 2891 | 2917 | 4117 | 4371 | 8746 | 7263 | 12212 | 10392 |
| 1539 | 1406 | 2226 | 1893 | 2236 | 2189 | 3914 | 3816 |
| 1705 | 1721 | 2010 | 1895 | 2886 | 3175 | 3770 | 3944 |
| 3356 | 4233 | 4121 | 4728 | 19322 | 16590 | 21306 | 20228 |
| 1923 | 1479 | 3107 | 1903 | 1849 | 1388 | 2943 | 2583 |
| 3732 | 3562 | 4729 | 4109 | 12283 | 9626 | 16311 | 12703 |
| 3392 | 2742 | 4227 | 5077 | 11648 | 8300 | 13049 | 11394 |
| 2494 | 2633 | 3277 | 3296 | 12641 | 12065 | 13195 | 13282 |
| 2689 | 2198 | 2955 | 3042 | 7671 | 6724 | 9485 | 7658 |
| 2738 | 2247 | 3259 | 2690 | 4812 | 4580 | 6581 | 5910 |
| 4916 | 3034 | 5165 | 3278 | 9127 | 6869 | 13553 | 9436 |
| 1696 | 1606 | 3408 | 2619 | 5435 | 3843 | 8474 | 6530 |
| 2379 | 2396 | 4073 | 3690 | 7976 | 7670 | 11627 | 10595 |
| 1762 | 1829 | 3100 | 3029 | 3372 | 2906 | 5796 | 5150 |
| 3137 | 2369 | 3202 | 3161 | 6341 | 5155 | 9333 | 7712 |
| 1359 | 1285 | 1737 | 1533 | 2081 | 1698 | 2788 | 2468 |
| 3152 | 2781 | 4028 | 3372 | 7098 | 6699 | 8579 | 9179 |
| 1862 | 1992 | 2533 | 2333 | 3625 | 4129 | 4709 | 5443 |
| 1682 | 1564 | 2532 | 2142 | 2637 | 2409 | 3975 | 3838 |


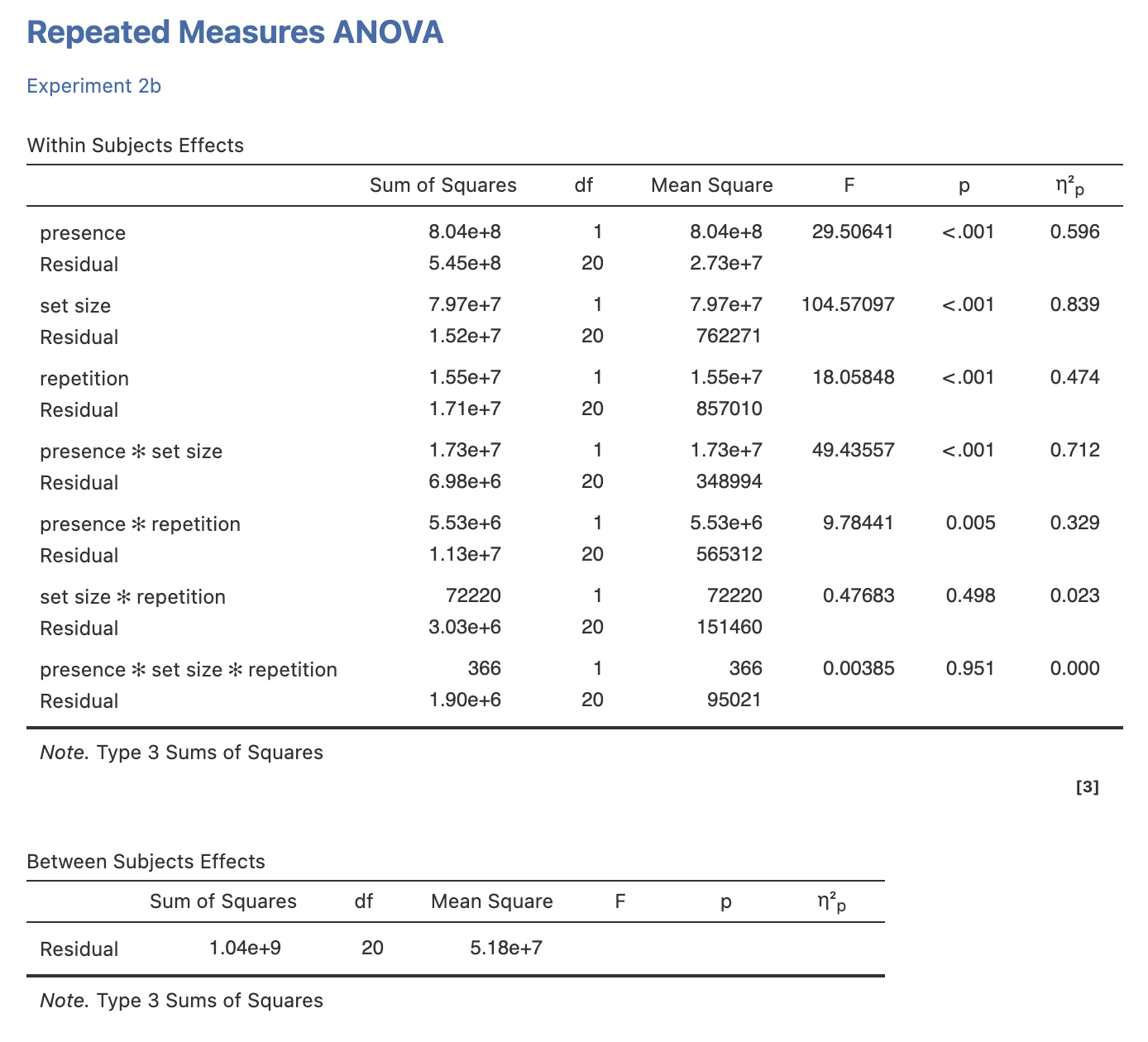


**Figure S3. RT analyses for Experiment 2b.**

**Table S4-1. RTs (ms) in Experiment 3a (noCue - noCue).**

| Target present | | | | Target absent | | | |
| --- | --- | --- | --- | --- | --- | --- | --- |
| Ss = 18 | | Ss = 36 | | Ss = 18 | | Ss = 36 | |
| Rep1 | Rep2 | Rep1 | Rep2 | Rep1 | Rep2 | Rep1 | Rep2 |
| 1197 | 1375 | 1749 | 1600 | 1846 | 2040 | 2300 | 2759 |
| 1694 | 1621 | 2361 | 2154 | 3805 | 3652 | 5272 | 4427 |
| 2346 | 1829 | 2746 | 2628 | 5638 | 4785 | 7620 | 6908 |
| 1564 | 1958 | 2716 | 2943 | 5442 | 5381 | 6142 | 5961 |
| 3381 | 2806 | 3813 | 3653 | 10941 | 8167 | 13145 | 10846 |
| 2583 | 2044 | 2313 | 2412 | 3979 | 3287 | 4356 | 4287 |
| 1561 | 1627 | 2181 | 1707 | 2695 | 2705 | 3812 | 3846 |
| 1529 | 1263 | 1581 | 1424 | 2031 | 1740 | 2491 | 2119 |
| 1731 | 1622 | 1881 | 867 | 1821 | 1490 | 1985 | 1644 |
| 2605 | 2514 | 2777 | 3212 | 9958 | 8441 | 12204 | 9323 |
| 2226 | 2718 | 3054 | 3886 | 8920 | 8591 | 11127 | 10101 |
| 2904 | 2673 | 4911 | 4030 | 6988 | 6881 | 12108 | 11291 |
| 1639 | 2032 | 2527 | 2817 | 3671 | 3410 | 5068 | 4448 |
| 1602 | 1522 | 1942 | 2558 | 2816 | 2357 | 3826 | 3462 |
| 1506 | 1389 | 2069 | 1981 | 3443 | 3435 | 5296 | 4904 |
| 1832 | 1672 | 2142 | 2089 | 3583 | 3215 | 5054 | 4278 |
| 2271 | 2035 | 2605 | 2336 | 5171 | 3874 | 6676 | 5218 |
| 1237 | 1160 | 1427 | 1355 | 1750 | 1612 | 2080 | 2075 |
| 1575 | 1136 | 1586 | 1600 | 2105 | 2058 | 2917 | 2888 |

**Table S4-2. RTs (ms) in Experiment 3a (noCue - Cue).**

| Target present | | | | Target absent | | | |
| --- | --- | --- | --- | --- | --- | --- | --- |
| Ss = 18 | | Ss = 36 | | Ss = 18 | | Ss = 36 | |
| Rep1 | Rep2 | Rep1 | Rep2 | Rep1 | Rep2 | Rep1 | Rep2 |
| 1240 | 1316 | 1591 | 1773 | 1772 | 2131 | 2217 | 2523 |
| 1463 | 1643 | 1893 | 1950 | 4109 | 3634 | 5097 | 4632 |
| 1698 | 1487 | 3093 | 1920 | 5695 | 5091 | 7986 | 7434 |
| 1968 | 2199 | 2976 | 3084 | 6002 | 4978 | 6284 | 6141 |
| 2635 | 2227 | 3438 | 3127 | 10724 | 8883 | 12436 | 11006 |
| 1745 | 1713 | 2418 | 1856 | 3820 | 3120 | 4070 | 4204 |
| 2000 | 1707 | 2155 | 2248 | 3017 | 2775 | 3780 | 3424 |
| 1173 | 1253 | 1919 | 1617 | 2281 | 1838 | 2656 | 2168 |
| 1273 | 1745 | 1408 | 1555 | 1724 | 1381 | 1715 | 1542 |
| 3342 | 2410 | 3081 | 2783 | 10261 | 8190 | 11525 | 10082 |
| 2381 | 2987 | 3725 | 3348 | 8613 | 7464 | 12516 | 10758 |
| 2222 | 2402 | 3697 | 3497 | 7546 | 6915 | 11224 | 10348 |
| 1914 | 1829 | 2359 | 2375 | 3467 | 3612 | 4625 | 4735 |
| 1405 | 1970 | 2581 | 1970 | 2808 | 2684 | 3695 | 3461 |
| 2188 | 1954 | 2195 | 1950 | 3553 | 3410 | 5023 | 4939 |
| 1549 | 1672 | 1907 | 1872 | 3446 | 3439 | 5181 | 4391 |
| 2045 | 2065 | 2552 | 2311 | 5643 | 3870 | 7075 | 5055 |
| 1480 | 1475 | 1503 | 1454 | 1616 | 1638 | 1992 | 2074 |
| 1236 | 1264 | 1777 | 1551 | 2007 | 2058 | 3123 | 2862 |


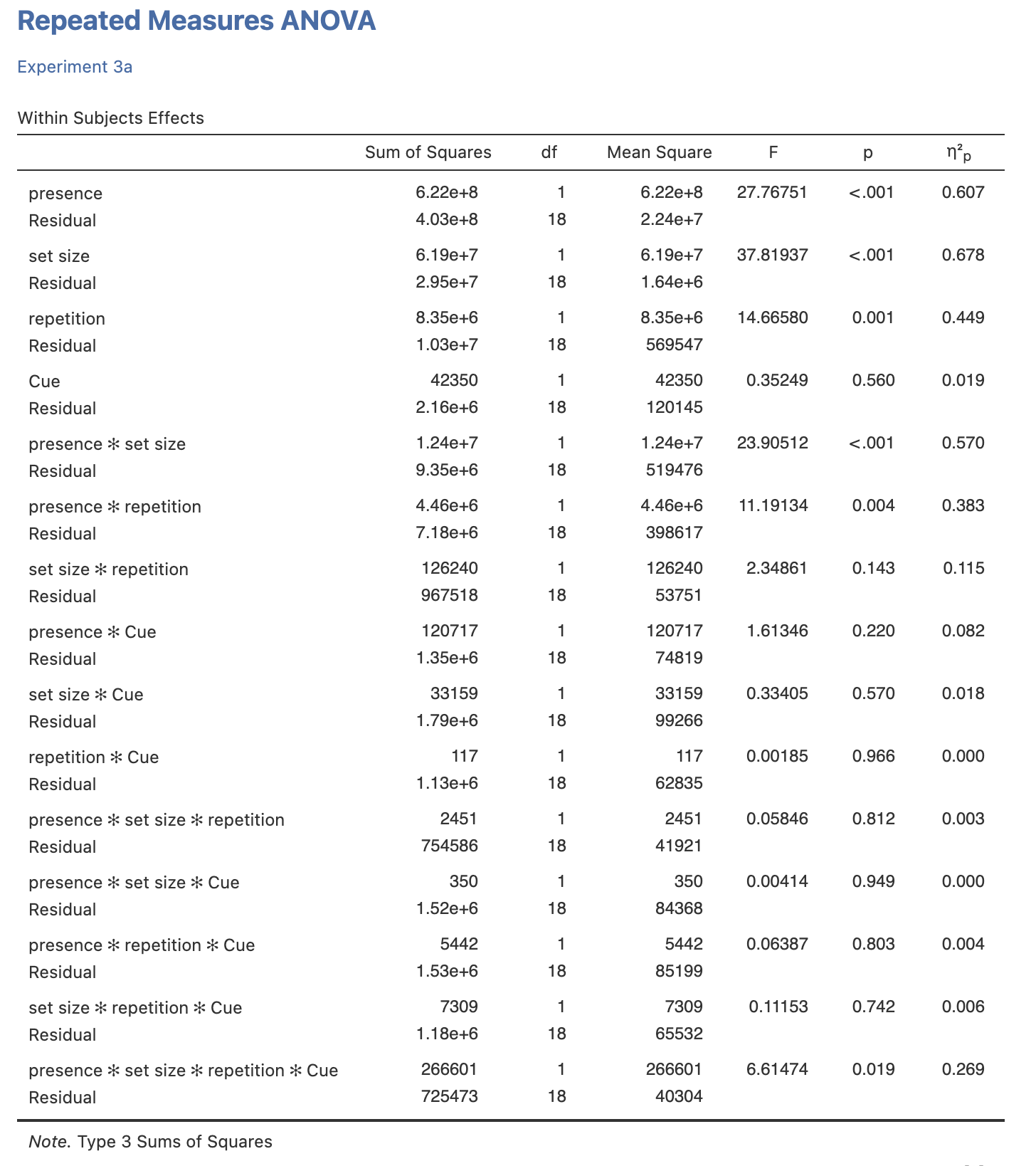


**Figure S4-1. RT analyses for Experiment 3a (Four-way ANOVA).**


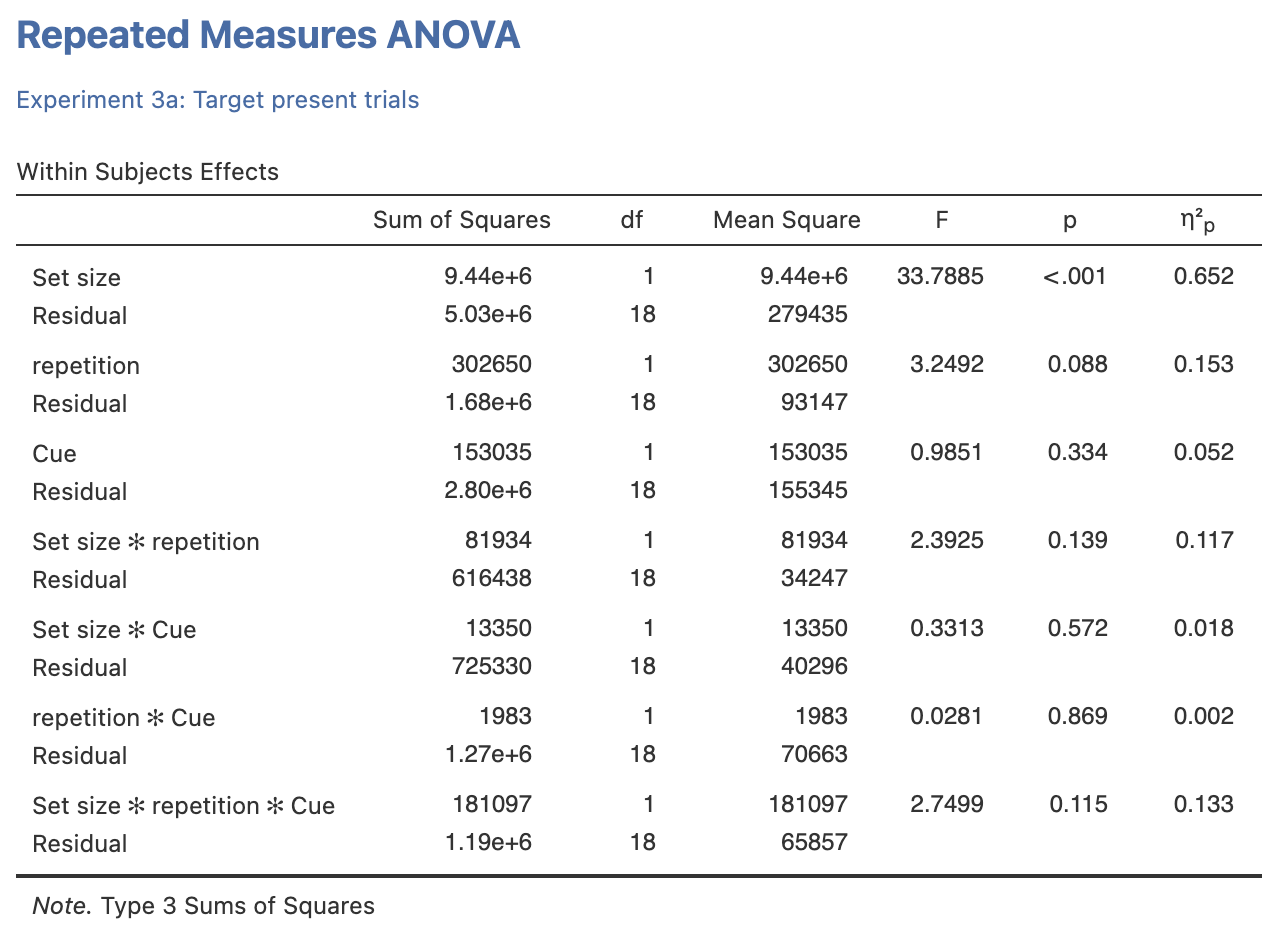


**Figure S4-2. RT analyses for Experiment 3a (Three-way ANOVA on target present trials).**


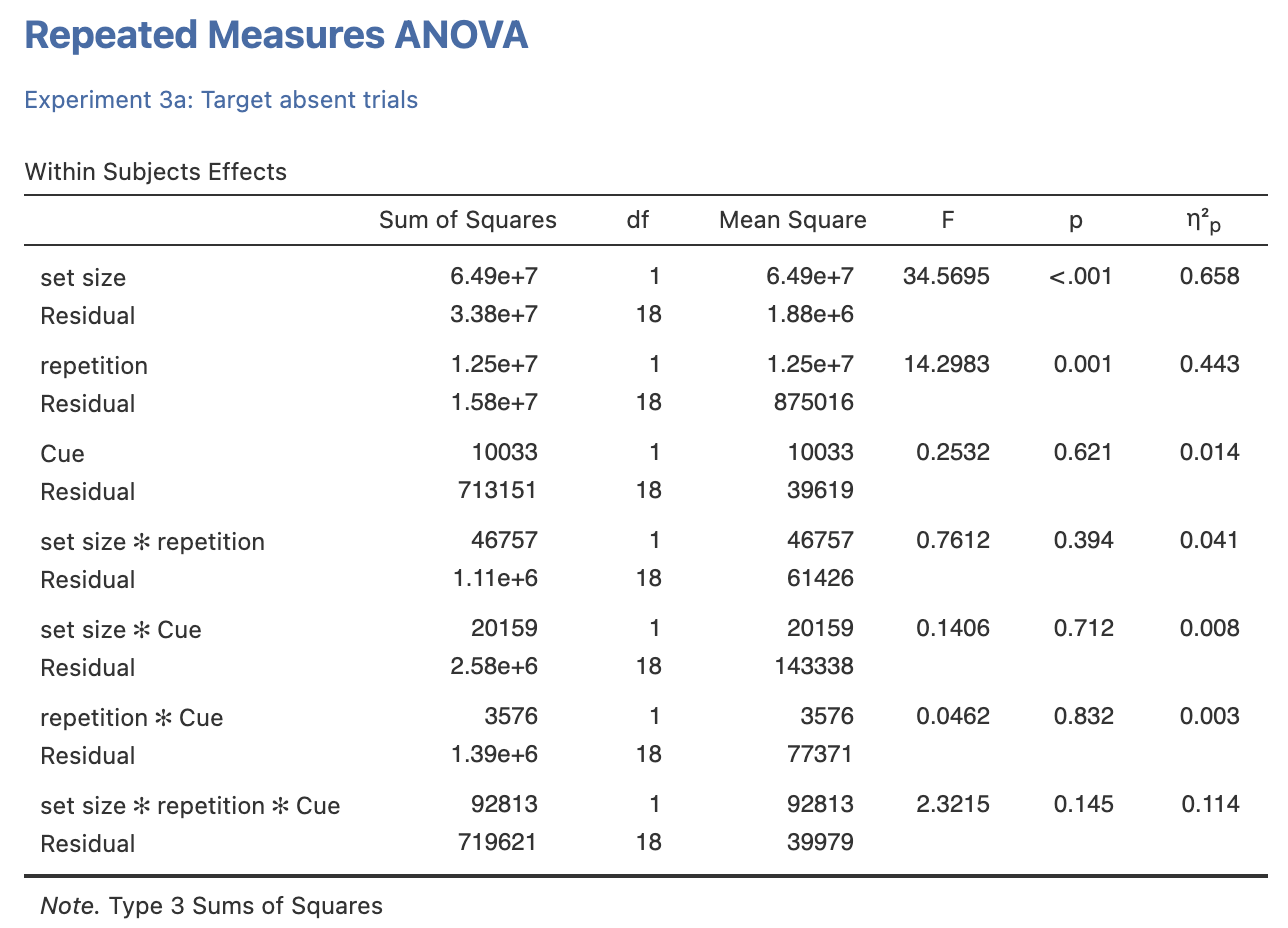


**Figure S4-3. RT analyses for Experiment 3a (Three-way ANOVA on target absent trials).**

**Table S5-1. RTs (ms) in Experiment 3b (noCue - noCue).**

| Target present | | | | Target absent | | | |
| --- | --- | --- | --- | --- | --- | --- | --- |
| Ss = 18 | | Ss = 36 | | Ss = 18 | | Ss = 36 | |
| Rep1 | Rep2 | Rep1 | Rep2 | Rep1 | Rep2 | Rep1 | Rep2 |
| 2777 | 2439 | 3990 | 4937 | 9282 | 6933 | 10977 | 7162 |
| 1907 | 1969 | 2926 | 2043 | 3503 | 3869 | 4502 | 4377 |
| 2389 | 1872 | 1928 | 2466 | 2971 | 3058 | 4190 | 3821 |
| 1612 | 1457 | 2115 | 2302 | 2593 | 2396 | 3702 | 3591 |
| 2100 | 1517 | 3215 | 2917 | 5413 | 4658 | 8121 | 6814 |
| 1583 | 1242 | 2305 | 2030 | 3147 | 2186 | 4673 | 3212 |
| 1503 | 1723 | 2153 | 1710 | 3444 | 3072 | 4980 | 4423 |
| 1920 | 1757 | 3012 | 2478 | 4008 | 3700 | 5805 | 5195 |
| 1689 | 1552 | 2268 | 2249 | 2983 | 2814 | 4053 | 3525 |
| 2076 | 2288 | 3533 | 3056 | 5304 | 3681 | 8223 | 5916 |
| 2120 | 1715 | 2444 | 2541 | 3229 | 2595 | 3631 | 3115 |
| 1408 | 1215 | 1975 | 1599 | 2251 | 2013 | 2557 | 2465 |
| 2193 | 2136 | 2853 | 2285 | 4288 | 4635 | 5967 | 5790 |
| 1633 | 1582 | 1779 | 2463 | 2987 | 2581 | 4180 | 3574 |
| 2211 | 2127 | 3097 | 2506 | 5137 | 5056 | 7331 | 7107 |
| 2612 | 2179 | 3654 | 3483 | 9586 | 6074 | 12170 | 7483 |
| 2644 | 2081 | 2700 | 2797 | 2752 | 2481 | 4039 | 3307 |
| 1321 | 1340 | 2375 | 2229 | 2415 | 2333 | 3836 | 3486 |
| 2769 | 2786 | 3022 | 4494 | 6249 | 6731 | 9216 | 8426 |

**Table S5-2. RTs (ms) in Experiment 3b (noCue - Cue).**

| Target present | | | | Target absent | | | |
| --- | --- | --- | --- | --- | --- | --- | --- |
| Ss = 18 | | Ss = 36 | | Ss = 18 | | Ss = 36 | |
| Rep1 | Rep2 | Rep1 | Rep2 | Rep1 | Rep2 | Rep1 | Rep2 |
| 3309 | 2574 | 4898 | 3856 | 9112 | 7357 | 11242 | 7886 |
| 2277 | 2221 | 3142 | 2540 | 3561 | 3918 | 4383 | 5207 |
| 2050 | 2450 | 2859 | 2309 | 2975 | 3080 | 4333 | 4173 |
| 2071 | 1649 | 1979 | 1915 | 2654 | 2484 | 3563 | 3889 |
| 2255 | 2014 | 3434 | 2552 | 5027 | 5199 | 7902 | 7209 |
| 1633 | 1796 | 2622 | 2020 | 3687 | 3198 | 4260 | 3986 |
| 1804 | 2120 | 2181 | 2108 | 3157 | 3071 | 4932 | 5152 |
| 1857 | 2037 | 2381 | 2482 | 4174 | 3455 | 6852 | 4934 |
| 1707 | 1494 | 2623 | 1943 | 3038 | 2618 | 3880 | 3566 |
| 1929 | 2273 | 3235 | 2657 | 5665 | 4627 | 9968 | 6782 |
| 1858 | 1882 | 1394 | 2068 | 3356 | 3022 | 3806 | 3574 |
| 1442 | 1503 | 2008 | 1698 | 2046 | 1994 | 2572 | 2451 |
| 2744 | 2380 | 2821 | 2452 | 4575 | 4290 | 5231 | 5373 |
| 1720 | 1533 | 2541 | 2636 | 2707 | 2523 | 4150 | 3583 |
| 2065 | 2201 | 2994 | 3118 | 5081 | 5972 | 7402 | 7689 |
| 2446 | 3235 | 3531 | 4616 | 8785 | 6910 | 10973 | 10027 |
| 2240 | 1604 | 2295 | 2020 | 3002 | 2320 | 3706 | 3437 |
| 1900 | 1702 | 2543 | 1958 | 2438 | 2459 | 3853 | 3306 |
| 2616 | 3402 | 3470 | 3228 | 7102 | 6978 | 8759 | 9661 |


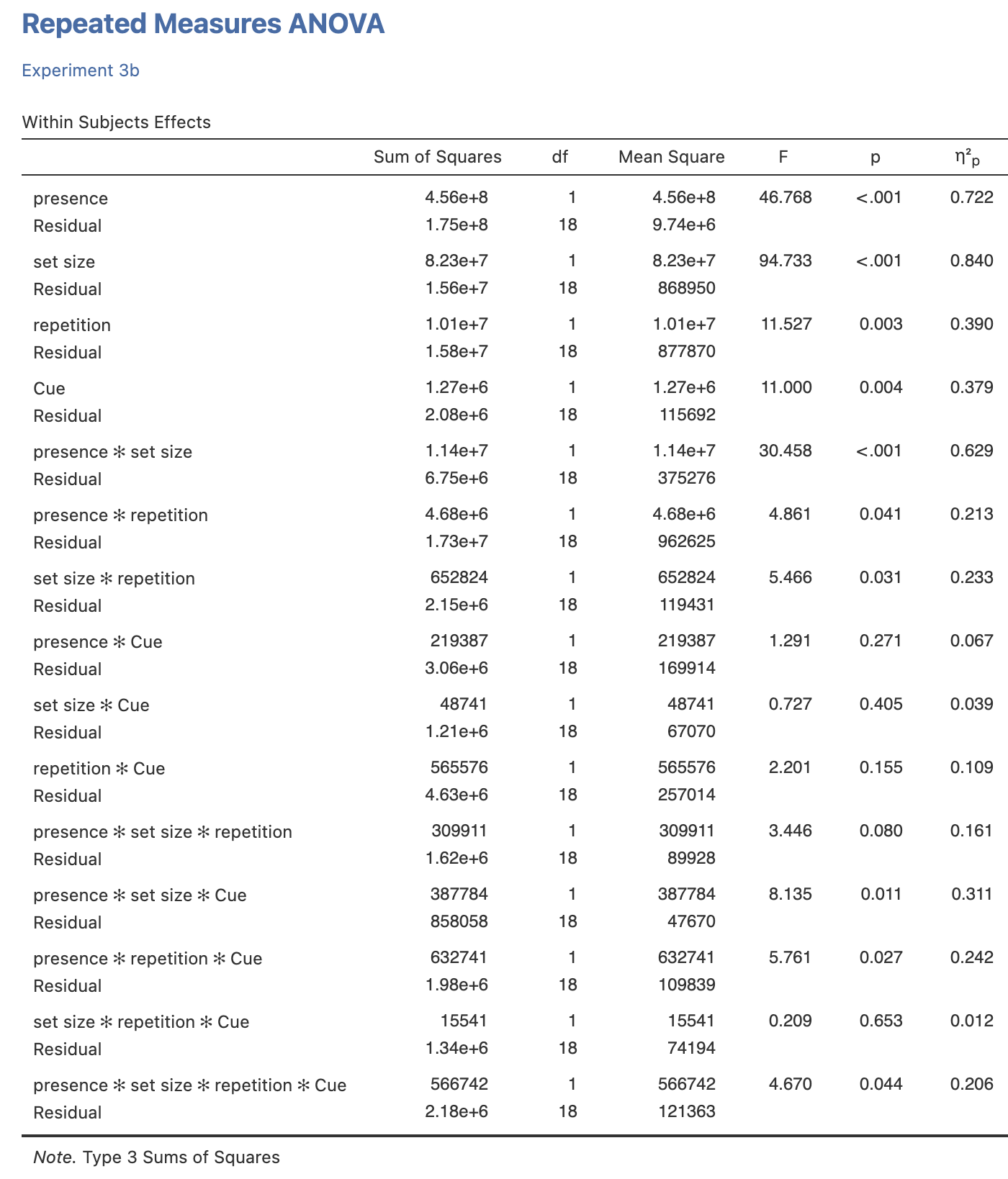


**Figure S5-1. RT analyses for Experiment 3b (Four-way ANOVA).**


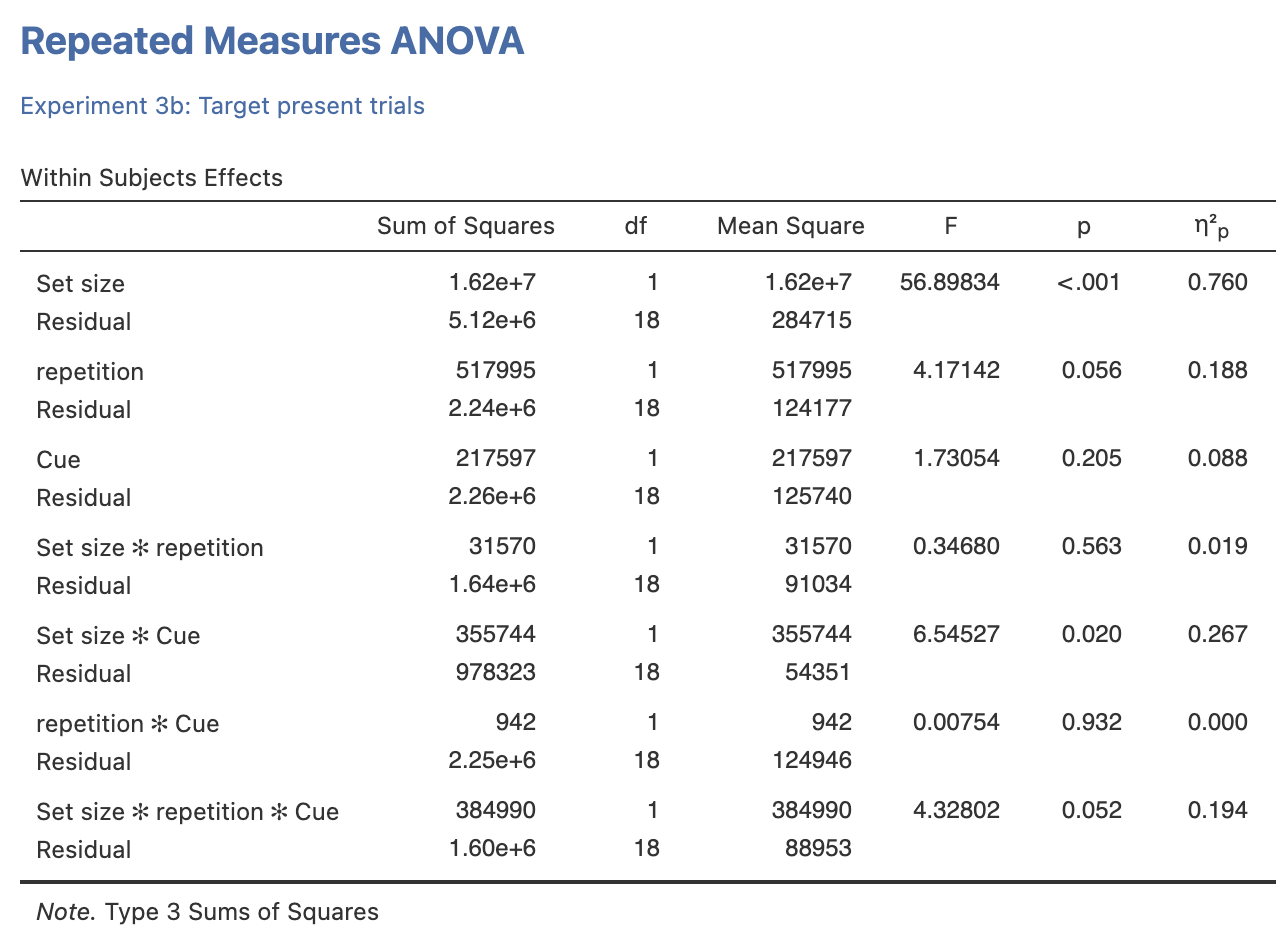


**Figure S5-2. RT analyses for Experiment 3b (Three-way ANOVA on target present trials).**


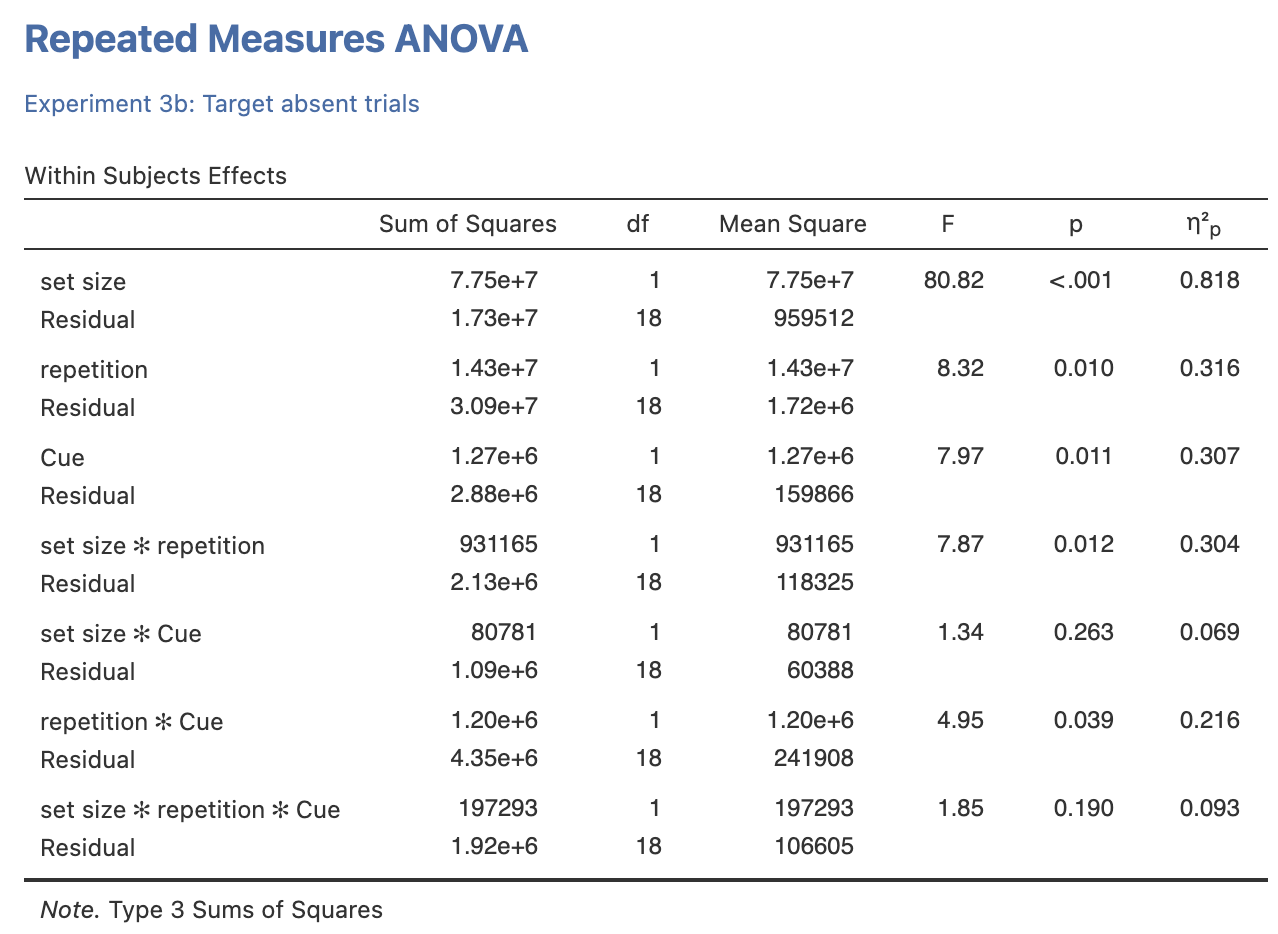


**Figure S5-3. RT analyses for Experiment 3b (Three-way ANOVA on target absent trials).**

**Table S6-1. RTs (ms) in Experiment 3c (noCue - noCue).**

| Target present | | | | Target absent | | | |
| --- | --- | --- | --- | --- | --- | --- | --- |
| Ss = 18 | | Ss = 36 | | Ss = 18 | | Ss = 36 | |
| Rep1 | Rep2 | Rep1 | Rep2 | Rep1 | Rep2 | Rep1 | Rep2 |
| 2692 | 2576 | 4236 | 3405 | 6876 | 6049 | 8231 | 7116 |
| 3805 | 3841 | 6169 | 5337 | 4890 | 6078 | 8160 | 8344 |
| 1689 | 1697 | 1810 | 1976 | 2578 | 2368 | 3142 | 3171 |
| 2050 | 2257 | 2741 | 2533 | 7579 | 5109 | 8771 | 6960 |
| 2591 | 2621 | 2722 | 1879 | 3474 | 2880 | 4684 | 3743 |
| 2677 | 3136 | 4907 | 4180 | 5606 | 4780 | 7036 | 6890 |
| 1204 | 1206 | 1761 | 1680 | 1758 | 1586 | 2425 | 2216 |
| 2026 | 2423 | 2640 | 2444 | 3899 | 3604 | 4727 | 4943 |
| 3147 | 2632 | 3940 | 3335 | 6797 | 6703 | 10495 | 8759 |
| 2096 | 2171 | 3317 | 3053 | 3567 | 3422 | 6009 | 6038 |
| 2001 | 1506 | 2190 | 1911 | 4442 | 2976 | 5937 | 4627 |
| 2380 | 2407 | 3262 | 2821 | 5743 | 4929 | 9143 | 6365 |
| 2084 | 1741 | 2370 | 2289 | 3366 | 3710 | 5003 | 5557 |
| 1829 | 1277 | 2161 | 1788 | 2699 | 2121 | 4324 | 3378 |
| 2564 | 2367 | 2813 | 2821 | 4482 | 4093 | 6213 | 5405 |
| 1729 | 1537 | 1920 | 1784 | 3340 | 3282 | 4984 | 4459 |
| 3815 | 3072 | 4105 | 3263 | 6078 | 5223 | 7069 | 6285 |
| 2411 | 1855 | 2165 | 2477 | 3318 | 3308 | 4474 | 3761 |
| 1837 | 2266 | 3150 | 2766 | 3833 | 2929 | 5610 | 4685 |

**Table S6-2. RTs (ms) in Experiment 3c (noCue - Cue).**

| Target present | | | | Target absent | | | |
| --- | --- | --- | --- | --- | --- | --- | --- |
| Ss = 18 | | Ss = 36 | | Ss = 18 | | Ss = 36 | |
| Rep1 | Rep2 | Rep1 | Rep2 | Rep1 | Rep2 | Rep1 | Rep2 |
| 2354 | 2761 | 3736 | 5236 | 6768 | 6288 | 8769 | 9468 |
| 1962 | 4342 | 4066 | 4918 | 5962 | 7380 | 8174 | 10515 |
| 1980 | 1888 | 2440 | 2313 | 2453 | 3173 | 3213 | 3786 |
| 2651 | 2194 | 4082 | 3095 | 7054 | 4611 | 9087 | 7558 |
| 1730 | 2249 | 2985 | 3562 | 2942 | 4073 | 5207 | 6066 |
| 2480 | 2993 | 4556 | 3868 | 6063 | 5998 | 7966 | 9613 |
| 1339 | 1591 | 1842 | 1674 | 1844 | 2225 | 2613 | 2523 |
| 2230 | 2274 | 2343 | 2854 | 3908 | 4738 | 5444 | 6711 |
| 3321 | 3190 | 4541 | 5163 | 7767 | 6718 | 10482 | 10066 |
| 2462 | 3119 | 3499 | 3851 | 3557 | 4779 | 6047 | 8310 |
| 1809 | 2035 | 3309 | 2180 | 4216 | 3569 | 6010 | 5624 |
| 2974 | 2761 | 3310 | 3204 | 5682 | 5035 | 8158 | 7987 |
| 1802 | 2494 | 2416 | 3602 | 3205 | 5864 | 4078 | 9305 |
| 1333 | 1618 | 2107 | 2673 | 2772 | 3383 | 4120 | 4670 |
| 2885 | 3301 | 3350 | 4284 | 5521 | 5329 | 6076 | 7467 |
| 2193 | 2067 | 2248 | 2232 | 3375 | 3799 | 4782 | 5918 |
| 3355 | 3422 | 4988 | 5324 | 6754 | 6713 | 7961 | 9191 |
| 2252 | 2134 | 2290 | 2500 | 3318 | 4086 | 4593 | 4980 |
| 1992 | 2309 | 3899 | 2298 | 3979 | 3653 | 6379 | 5821 |


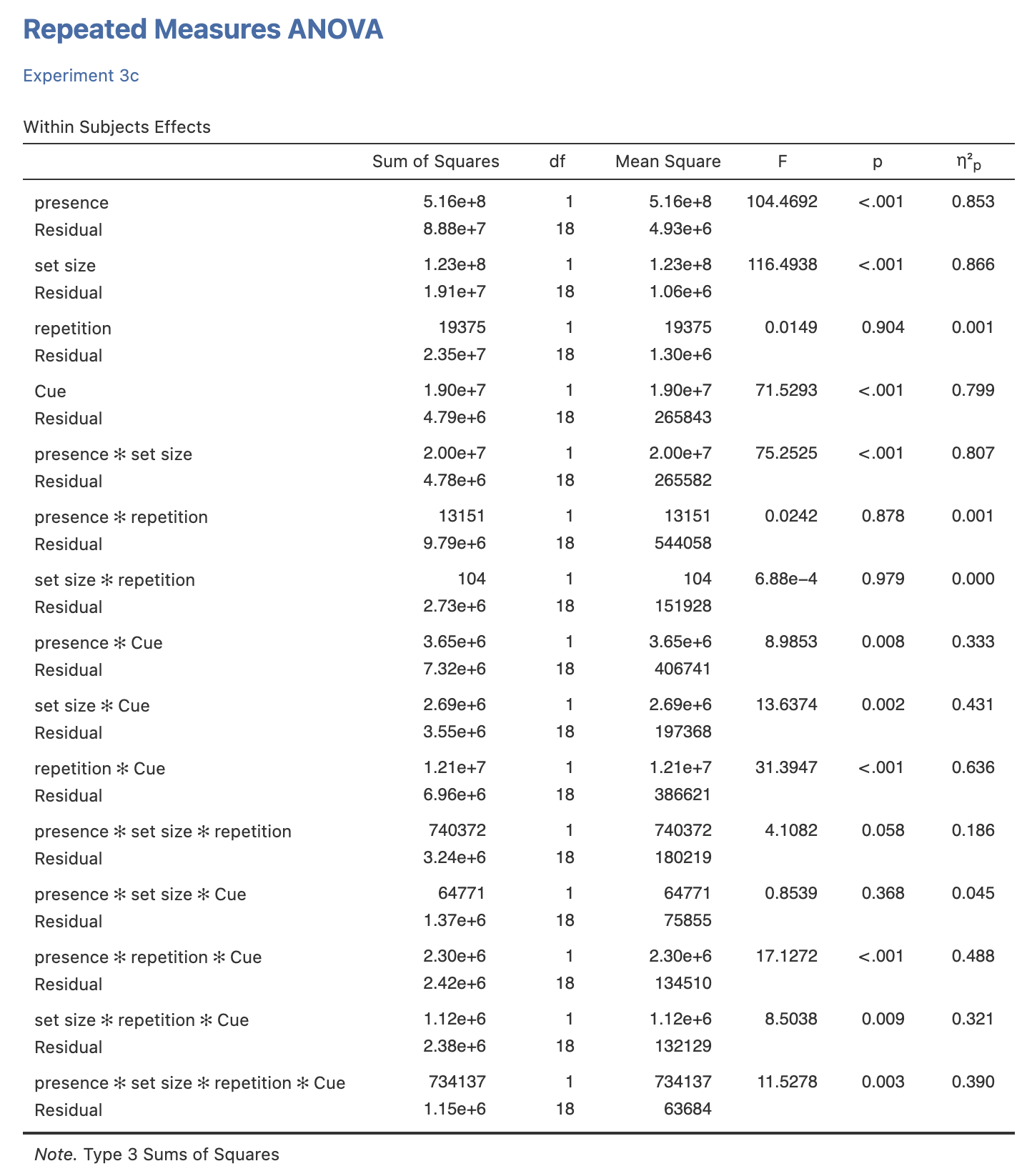


**Figure S6-1. RT analyses for Experiment 3c (Four-way ANOVA).**


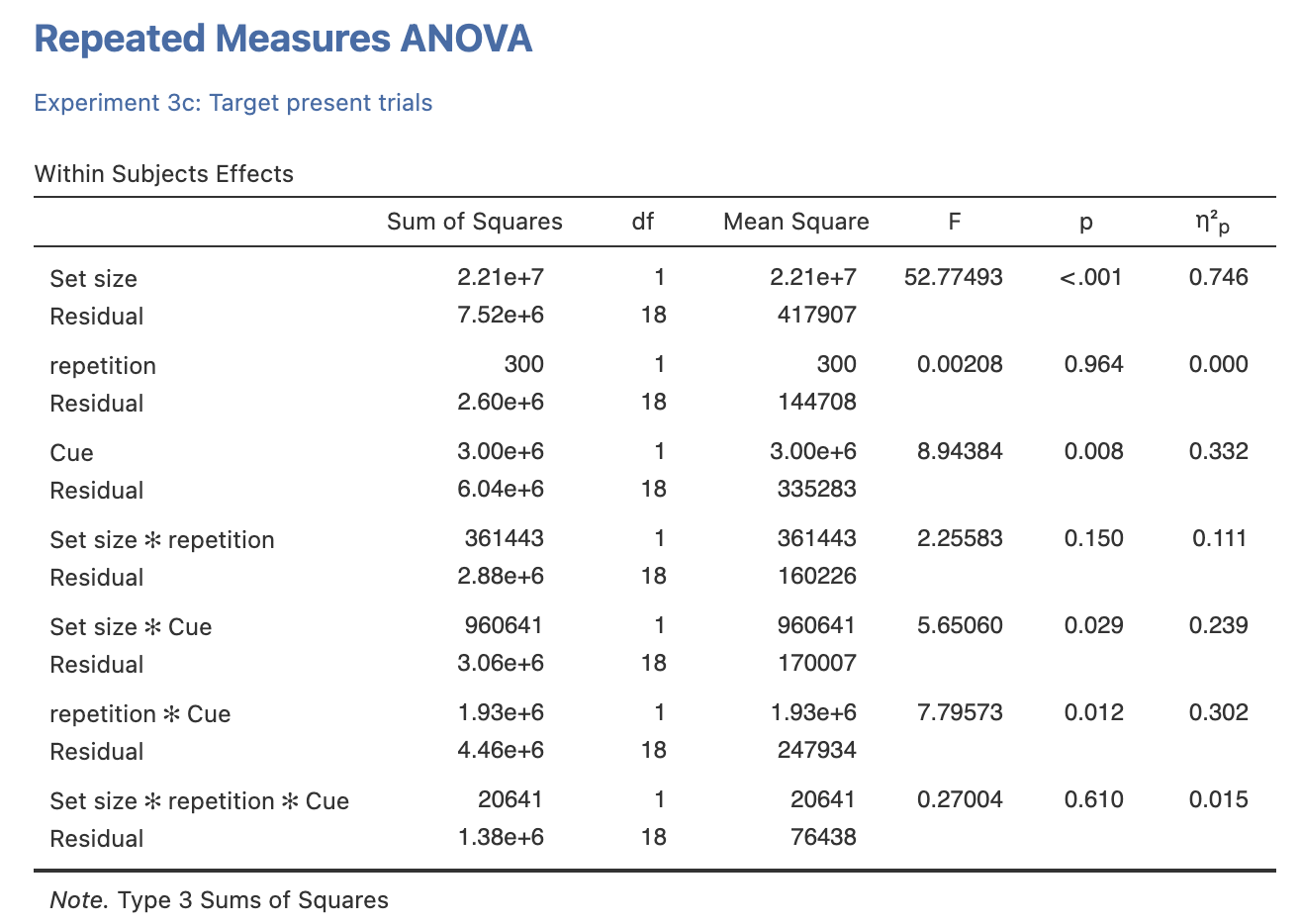


**Figure S6-2. RT analyses for Experiment 3c (Three-way ANOVA on target present trials).**


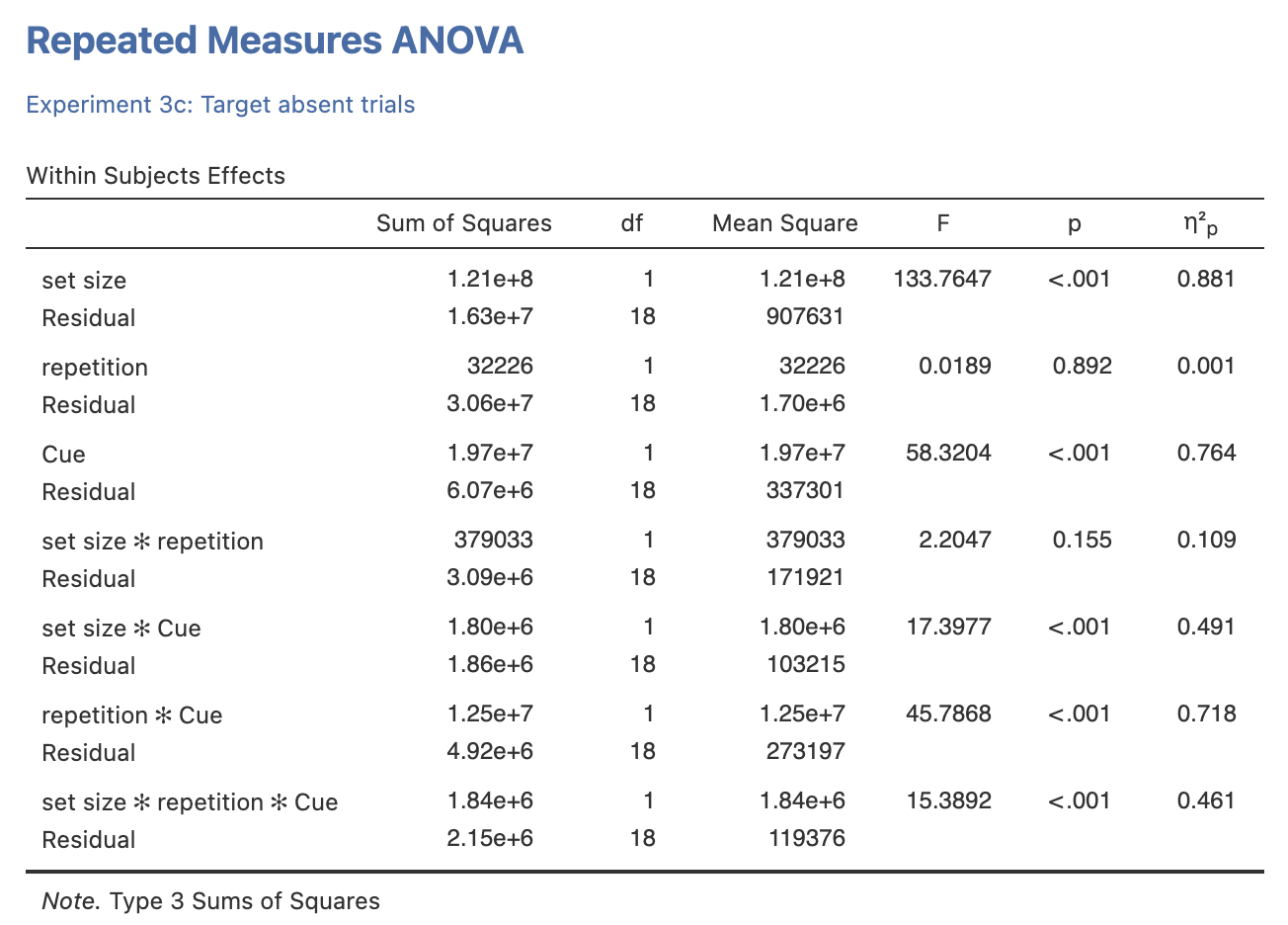


**Figure S6-3. RT analyses for Experiment 3c (Three-way ANOVA on target absent trials).**
